# Modular Synthesis of Heparin-Related Tetra-, Hexa- and Octasaccharides with Differential *O*-6 Protections: Programming for Regiodefined 6-*O*-Modifications

**DOI:** 10.3390/molecules20046167

**Published:** 2015-04-09

**Authors:** Marek Baráth, Steen U. Hansen, Charlotte E. Dalton, Gordon C. Jayson, Gavin J. Miller, John M. Gardiner

**Affiliations:** 1 Manchester Institute of Biotechnology and the School of Chemistry, 131 Princess Street, The University of Manchester, Manchester M1 7DN, UK; 2 Institute of Cancer Sciences, Christie Hospital and University of Manchester, Wilmslow Road, Manchester M20 4BX, UK

**Keywords:** heparin, oligosaccharide, iduronate, GAG mimetic

## Abstract

Heparin and heparan sulphate (H/HS) are important members of the glycosaminoglycan family of sugars that regulate a substantial number of biological processes. Such biological promiscuity is underpinned by hetereogeneity in their molecular structure. The degree of *O*-sulfation, particularly at the 6-position of constituent d-GlcN units, is believed to play a role in modulating the effects of such sequences. Synthetic chemistry is essential to be able to extend the diversity of HS-like fragments with defined molecular structure, and particularly to deconvolute the biological significance of modifications at O6. Here we report a synthetic approach to a small matrix of protected heparin-type oligosaccharides, containing orthogonal d-GlcN *O*-6 protecting groups at programmed positions along the chain, facilitating access towards programmed modifications at specific sites, relevant to sulfation or future mimetics.

## 1. Introduction

**Figure 1:**
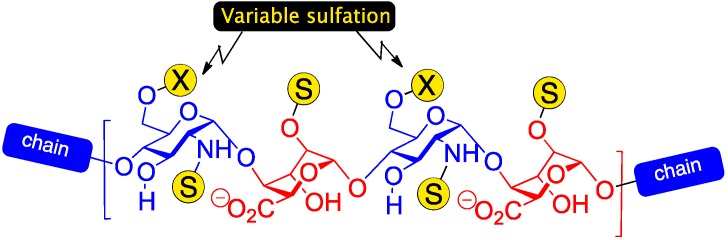
Higher sulfation heparin-type region with varying levels of GlcN *O*-6 sulfation.

Heparin and heparan sulphate (H/HS) are ubiquitous biological oligosaccharides which regulate many important signalling processes, especially within the extracellular matrix [1,2,3,4,5]. H/HS oligosaccharides consist of repeating glucosamine and uronic acid monomers and are highly polydisperse, varying not only in the backbone constitution (l-iduronic and/or d-glucuronic acid residues) and length (including the biologically effective region), but also in a variety of sulfation patterns. Sulfation is obligatory on *O*-2, where l-IdoA is installed, but can be present on combinations of glucosamine *N* and *O*-6 sites. In native sequences, sulfation is typically organized into high- or lower-sulfation domains, and, along with the ratio of l-IdoA/D-GlcA, correlates to the heparin/heparan sulfate designation. Sequences which contain l-iduronic acid have attracted particular interest due to the evidence of many key bio-effector sequences being l-IdoA enriched (higher-sulphated, l-idoA heparin-like structure shown in Figure 1). Understanding structure/function relationships of H/HS chains that modulate such processes is a major challenge in chemical biology.

The extensive microhetereogeneity of these oligosaccharides ensures that methods for synthesis of diverse, defined H/HS fragments are central to revealing structure-functional information in their chemical biology [6,7] and synthetic interest in solution, solid phase and utilizing enzymatic methods has been expanding [8,9,10,11,12,13,14,15,16,17,18,19,20,21].

**Figure 2:**
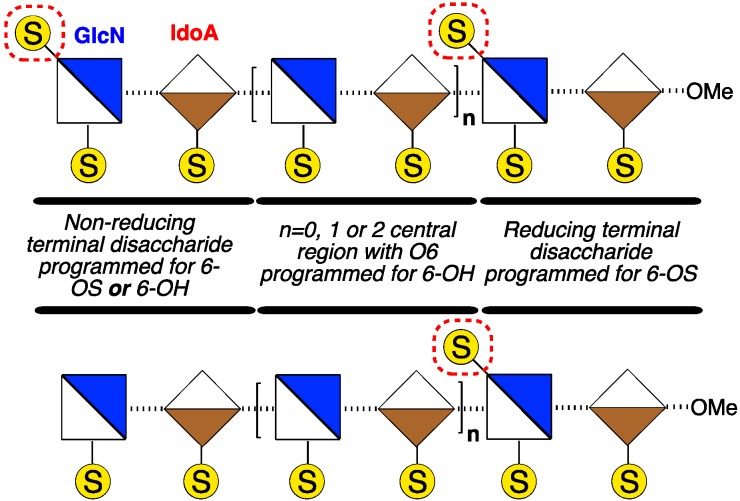
Strategic sequences with reducing terminus 6-*O*-sulfated (6-OS), central sequence de-6-*O*-sulfated (6-OH) and non-reducing terminus programmable for 6-OS or 6-OH.

Homogenous synthetic *O*-6-sulfation levels have been shown to affect the binding preferences of GAGs to target proteins [7,22,23], and recent examples have evidenced that even disaccharides can exhibit differential recognition based on sulfation patterns [24,25]. It is not yet clear how such binding effects translate to biological effects, nor how biological effects may be affected by defined regioselectivity of *O*-6 sulfation. There is, however, significant evidence that the overall level of glucosamine-6-*O*-sulfation, or de-6-*O*-sulfation, of D-GlcN, (Figure 1) is of central importance to effecting differential H/HS biological signalling events mediated by several HS-dependent cytokines (including CXCL8 [26,27], CXCL12 [28], FGF2 [29,30,31] and VEGF [28,30,31]). Many biological studies have employed enzymatically-generated heterogeneous materials as such native isolation only provides access to heterogeneous mixtures, differentiated by average sulfation levels. Interest in medium length structurally-defined heparanoids has been emboldened by the success of fondaparinux and activity of recently-reported longer synthetic mixed heparins [32]. This strongly suggests that evaluating programmed site-specific sulfation diversity is a high value target area. More ready availability of *O*-6 regiochemically differentiated fragments to underpin access towards such systems is required, and of particular utility would be medium length targets where terminal sulfate clustering or separation could be delivered (Figure 2).

**Figure 3:**
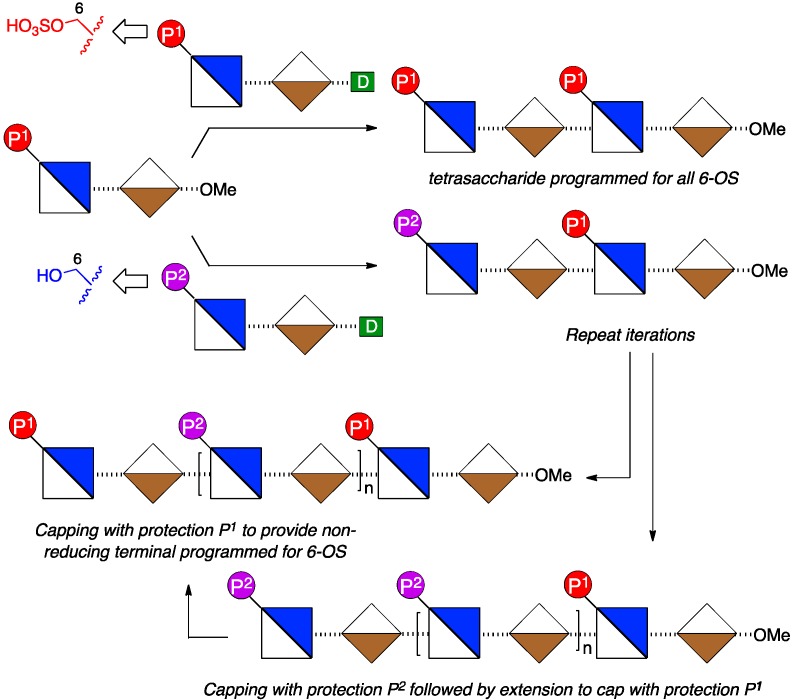
Strategy with fixed reducing terminal O6 protection to provide a matrix of oligosaccharides with single programmed reducing terminal unit or both terminal units programmed for 6-OS by choice of protecting group (d = glycosyl donor group).

To access such a series of mono or bis-terminus-modified oligosaccharides requires application of two types of disaccharide donor module, bearing orthogonal protections of *O*-6 groups to provide correlation to sites for ultimate 6-*O*-de-sulfation or 6-*O*-sulfation respectively, in any target H/HS sequences (Figure 3). 

This, combined with a single reducing terminal disaccharide module could then be employed to access a ladder of all mono- and bis-terminal unit differentiated oligosaccharides.

## 2. Results and Discussion

**Figure 4:**
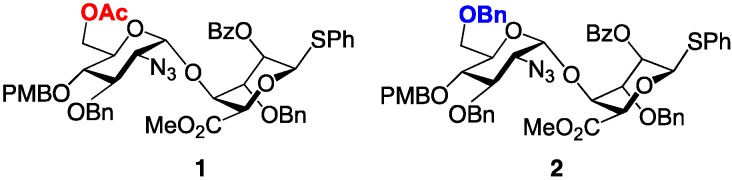
Disaccharide modules **1** and **2** with alternate d-GlcN 6-position protecting groups, programmed for access to 6-OH/6-OS final compounds.

As part of a program to develop efficient and scalable access to a variety of H/HS oligosaccharide fragments, we have developed effective methods to access heparin-like per-6-*O*-sufated [33] and per-6-*O*-desulfated [30,34,35] species. These syntheses underscore the efficacy of d-GlcN-l-IdoA-SPh donors **1** and **2** as efficient glycosyl building blocks in the construction of homogeneously *O*-6-derivatized oligomers up to dodecasaccharide. The d-GlcN 6-OH protecting groups (OBn or OAc) determine the fate of *O*-6 (either OBn = 6-OH or OAc = 6-OS), through common sulfation and deprotection steps (Figure 4). Here we extend this work to employ a modular approach to prepare protected H/HS-like precursor fragments containing regio-differentiated protections of sequence-specific O-6 sites, relevant to different H/HS fragments with programmed site-specific sulfations, or for the introduction of other region-defined mimetics. We demonstrate application of this to deliver five novel single-terminal and double-terminal differentiated tetra-, hexa- and octasaccharides across this matrix.

**Scheme 1:**
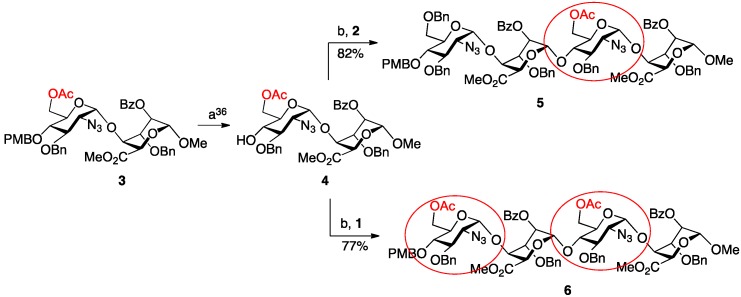
Synthesis of terminally-differentiated tetrasaccharides. (**a**) Ceric ammonium nitrate (CAN), MeCN/H_2_O; (**b**) *N*-iodosuccinimide (NIS), AgOTf, DCM.

The homologation matrix for all oligosaccharides thus began with OMe-capped disaccharide **3**, the gram scale synthesis of which we have previously reported [36]. Ceric ammonium nitrate mediated removal of the PMB group provided the required novel disaccharide acceptor **4** in high yield (Scheme 1). 

This material embeds *O*-6 protection in the reducing terminal disaccharide unit and thus allies deprotection and sulfation of d-GlcN *O*-6 in any derived oligosaccharide targets (in addition to the obligatory *O*-2 sulfation of the l-iduronic acid residue), whilst enabling potential controlled modification for new regiodefined mimetics.

Disaccharide **4** was then employed to illustrate parallel synthesis of tetra- and hexasaccharides, towards a target octasaccharide with double terminal *O*-6 acylated units. The first cycle of iteration illustrated use of acceptor **4** in two separate glycosylation reactions, firstly with donor module **1** (to install a second 6-OAc group) and secondly with donor **2** (to install a 6-OBn protecting group). Both glycosylations proceeded in good to excellent yields, affording the two new alpha-linked tetrasaccharides **5** and **6** in 82% and 77% yields, respectively.

To progress towards the next homologation targets with the same alternatives of non-reducing 6-OAc or 6-OBn units, tetrasaccharide **5** was subjected to the same two parallel glycosylations, again using either of the two disaccharide donor modules, **1** or **2**. Thus, **5** was first deprotected at the non-reducing end 4-position with CAN and then separately glycosylated with **1** and **2** to yield hexasaccharides **7** and **8** in very good to excellent yields (70% for **7**, 96% for **8**). This afforded the second tier of species with different *O*-6 protecting group patterns, encoding accessibility to H/HS species with d-GlcN 6OS-6OH-6OS or 6OH-6OH-6OS sulfations.

**Scheme 2:**
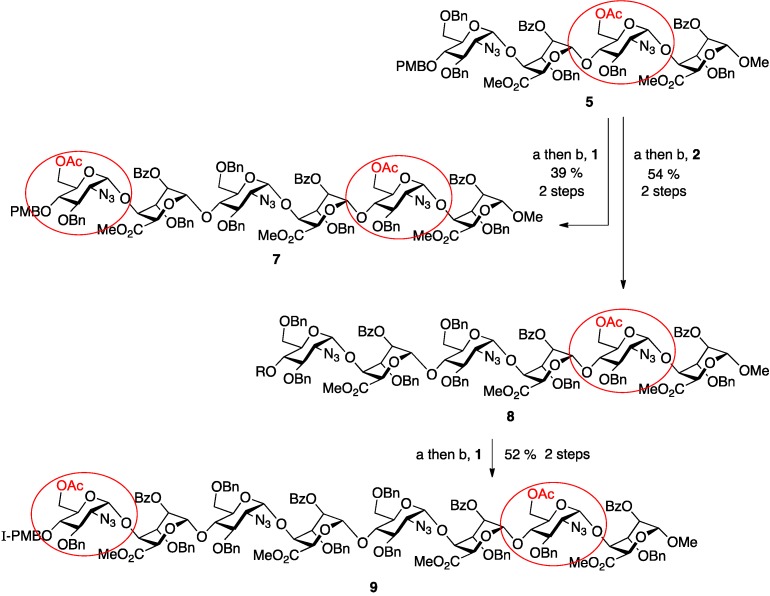
Synthesis of ladder of mono- or bis-terminally-differentiated hexasaccharides **7** and **8** and octasaccharide **9**. (**a**) CAN, MeCN/H_2_O; (**b**) NIS, AgOTf, DCM.

Finally, the mono-6-OAc hexasaccharide **8** was elaborated by a further cycle of iteration to octasaccharide **9**, providing a third oligosaccharide length with differentiated *O*-6 protecting groups encoding for ultimate sulfation at both the non-reducing and reducing termini. This iteration proceeded with similar efficiency to the prior cycles, with **8** deprotected via the established conditions in 78% yield and the resulting acceptor hexasaccharide glycosylated with iterative donor **1** to afford **9** in 66% yield (Scheme 2). 

Of note during this final iteration was the need to raise the number of molar equivalents of glycosylation activator, NIS, from 5 (in prior iterations), to 7 for formation of **9**. This resulted in effective glycosylation, but also a concomitant iodination of the electron rich PMB ring, as observed by ESI-MS (*m*/*z* = 1612.0377, 100%, z = 2 and no evidence of the non-iodinated species). This is a useful methodological note for SPh/NIS mediated glycosylations of substrates containing electron rich aromatic rings, but does not detract from the overall utility of the method, as the iodinated PMB group would necessarily be subsequently removed, either for any further iteration, or under final oligosaccharide deprotection procedures.

During these iterative steps both the disaccharide donors **1** or **2**, delivered glycosylation yields close to or above 70%, though in general the 6-OAc systems performed to give slightly lower yields than the 6-OBn analogues. This result is in keeping with our [33,35] and others [19,20] previously established routes using 6-*O*-ether/acetal *vs.* 6-*O*-ester type disaccharide building blocks to effect oligosaccharide syntheses.

**Figure 5:**
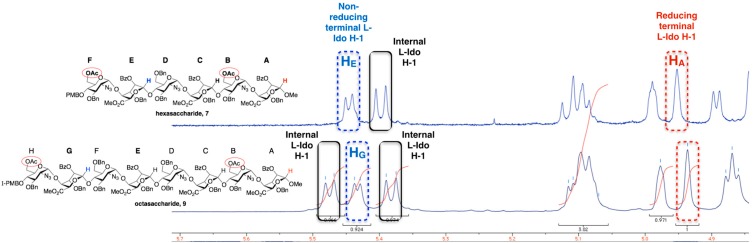
^1^H-NMR signals (400 MHz) for iduronate anomeric regions of hexasaccharide **7** and octasaccharide **9**.

For hexa- and octasacccharide systems **7** and **9**, ^1^H-NMR data indicated that all the l-iduronate unit conformations are dependent on location in the sequence. The ^3^*J*_H1-H2_ coupling constants are <6.0 Hz for all l-IdoA residues of **7** and **9** (Figure 5). Figure 5 illustrates a clear resolution of each constituent l-IdoA H_1_ signal allowing the result of an iterative oligosaccharide homologation process (*i.e*., transformation of **7** to **9**) to be readily correlated with such clear spectral dispersion. This dispersion also facilitates conformational analysis, the differential couplings for the reducing terminal l-IdoA H_1_ compared to the internal and non-reducing terminal l-IdoA H_1_ signals is consistent with the former being predominantly ^1^C_4_, whilst the larger couplings for the remaining l-IdoA units would be consistent with more significantly contributions from the ^2^S_o_ skew-boat [14,37].

## 3. Experimental Section

### General Information

Reagents and solvents were purchased from Sigma-Aldrich (Gillingham, UK) or Alfa Aesar (Heysham, UK). All reactions were carried out under an atmosphere of dry nitrogen, unless mentioned otherwise. ^1^H-NMR spectra were recorded on a Bruker Advance Ultrashield DPX 400 (400 MHz) and ^13^C-NMR spectra were recorded at 100 MHz on Bruker 300 and 400 DPX spectrometer. Melting points were obtained using a Stuart Scientific SMP1 melting point apparatus. Low-resolution mass spectra were recorded on a Micro Mass Trio 200 spectrometer (Wilmslow, UK) while high-resolution mass spectra were measured on a Kratos Concept IS spectrometer (Manchester, UK). Elemental analyses were performed using a Thermo Flash 2000 Organic Elemental Analyzer (ThermoFisher scientific, Warrington, UK) for CHN analysis. Flash chromatography was conducted using Merck (Nottingham, UK) silica gel 60 (particle size 40–60 μm). Analytical TLC were performed on Merck 60 F_254_ aluminium-backed plates containing a 254 nm fluorescent indicator. Optical rotations were measured at 589 nm in a 1 dm cell using an Optical Activity AA1000 polarimeter.

*Methyl 6-O-acetyl-2-azido-3-O-benzyl-2-deoxy-α-d-glucopyranosyl-(1→4)-(methyl 2-O-benzoyl-3-O-benzyl-α-l-idopyranoside) uronate* (**4**). Ceric (IV) ammonium nitrate (770 mg, 1.4 mmol) was added to a solution of **3** (600 mg, 0.7 mmol) in acetonitrile and water (17 mL, 8:1, *v*/*v*). The mixture was stirred for 1 h at ambient temperature and then diluted with DCM (150 mL), washed with saturated aq. NaHCO_3_ (100 mL) and saturated aqueous NaCl (50 mL). The organic phase was then dried over MgSO_4_ and solvent removed *in vacuo*. The product was purified by column chromatography (EtOAc/hexane 1:3), yielding **4** (470 mg, 0.64 mmol, 91%) as a foam. *R_f_* 0.31 (EtOAc/hexane 2:3); ${\left[\alpha \right]}_{\text{D}}^{20}$ = +8.6 (*c* = 0.26, CH_2_Cl_2_); ^1^H-NMR (400 MHz; CDCl_3_) δ 8.18–8.15 (m, 2H, Ar*H*), 7.52–7.17 (m, 13H, Ar*H*), 5.14–5.13 (brs, 1H, H_2IdoA_), 5.11–5.10 (brs, 1H, H_1IdoA_), 4.94 (d, 1H, *J* = 11.9 Hz, C*H*_2_Ph), 4.86 (d, *J* = 2.0 Hz, 1H, H_5IdoA_), 4.77 (d, 1H, *J* = 11.9 Hz, C*H*_2_Ph), 4.67 (d, *J* = 3.4 Hz, 1H, H_1GlcN_), 4.64 (dd, *J* = 12.6, 2.8 Hz, 1H, H_6aGlcN_), 4.29 (d, 1H, *J* = 10.6 Hz, C*H*_2_Ph), 4.17 (dd, *J* = 12.5, 2.2 Hz, 1H, H_6bGlcN_), 4.16–4.12 (m, 1H, H_4IdoA_), 4.03–4.00 (m, 3H, C*H*_2_Ph, H_3IdoA_), 3.46 (dt, *J* = 10.0, 2.4 Hz, 1H, H_5GlcN_), 3.81 (s, 3H, C(O)OC*H*_3_), 3.51 (s, 3H, OC*H*_3_), 3.45 (dd, *J* = 10.0, 8.8 Hz, 1H, H_4GlcN_), 3.33 (dd, *J* = 10.0, 9.0 Hz, 1H, H_3GlcN_), 3.18 (dd, *J* = 10.1, 3.6 Hz, 1H, H_2GlcN_), 2.10 (s, 3H, C(O)CH_3_); ^13^C-NMR (100 MHz; CDCl_3_) δ 172.3, 169.8, 165.6, 137.9, 137.5, 133.5, 130.1, 129.7, 128.9, 128.6, 128.6, 128.3, 128.1, 128.0, 100.5, 99.7, 79.3, 76.0, 75.0, 72.8, 72.6, 71.3, 70.4, 67.9, 67.1, 63.3, 62.7, 56.4, 52.5, 21.0; HRMS (FT MS NSI^+^) *m/z* calcd for C_37_H_45_N_4_O_13_ [M+NH_4_]^+^ 753.2978, found 753.2980.

*Methyl (2-azido-3,6-di-O-benzyl-2-deoxy-4-O-p-methoxybenzyl-α-d-glucopyranosyl-(1→4)-(methyl 2-O-benzoyl-3-O-benzyl-α-l-idopyranosyl) uronate)-(1→4)-6-O-acetyl-2-azido-3-O-benzyl-2-deoxy-α-d-glucopyranosyl-(1→4)-(methyl 2-O-benzoyl-3-O-benzyl-α-l-idopyranoside) uronate* (**5**). Acceptor **4** (770 mg, 1.05 mmol) and donor **2** (1.4 g, 1.42 mmol) were mixed together, evaporated from dry toluene (3 × 20 mL) and dried under high vacuum for 1 h. The foam was dissolved in dry DCM (20 mL) and powdered molecular sieves (4 Å, 650 mg) added. The mixture was cooled to 0 °C and NIS (1.18 g, 5.25 mmol) added. The mixture was stirred for a further 15 min at this temperature and a catalytic amount (small spatula tip) of AgOTf was then added. The mixture was kept under nitrogen at 0 °C for another 30 min and was then quenched by addition of saturated aqueous Na_2_S_2_O_3_ and saturated aqueous NaHCO_3_ (10 mL, 1:1, *v*/*v*). The suspension was filtered through Celite^®^, the phases separated and the organic layer washed with saturated aqueous NaCl (10 mL). The organic phase was then dried over MgSO_4_ and solvent removed *in vacuo*. The mixture was purified by column chromatography (EtOAc/hexane, 1:4→2:3), yielding **5** (1.3 g, 0.81 mmol, 77%). *R_f_* 0.31 (EtOAc/hexane 1:2); ^1^H-NMR (400 MHz; CDCl_3_) δ 8.14–8.09 (m, 4H, Bz-Ar*H*), 7.47–7.13 (m, 33H, Ar*H*), 7.07 (d, 1H, *J* = 8.7 Hz, PMB), 6.84 (d, 1H, *J* = 8.7 Hz, PMB), 5.46 (d, 1H, *J* = 4.8 Hz, H_1'IdoA_), 5.18–5.16 (m, 1H, H_2'IdoA_), 5.07 (brs, 1H, H_1IdoA_), 5.05 (brs, 1H, H_2IdoA_), 4.99 (d, 1H, *J* = 3.5 Hz, H_1GlcN_), 4.91 (d, 1H, *J* = 11.8 Hz, C*H*_2_Ph), 4.78–4.74 (m, 4H, H_5’IdoA_, H_5IdoA_, 2 × C*H*_2_Ph), 4.67–4.36 (m, 10H, H_1GlcN_, H_4’IdoA_, H_4’IdoA_, 7 × C*H*_2_Ph), 4.29–4.27 (m, 2H, C*H*_2_Ph), 4.13–4.06 (m, 2H, H_3’IdoA_, H_3IdoA_), 3.93–3.92 (m, 1H, H_5’GlcN_), 3.88–3.80 (s, 3H, PMBOC*H*_3_), 3.76–3.59 (m, 12H, H_3’GlcN_, H_3’GlcN_, H_4’GlcN_, H_4’GlcN_, H_5GlcN_, 2 × H_6’GlcN_, 2 × H_6GlcN_, OC*H*_3_), 3.47 (s, 6H, C(O)OC*H*_3_), 3.24 (dd, 1H, *J* = 10.1, 3.6 Hz, H_2’GlcN_), 3.19–3.15 (m, 1H, H_2GlcN_), 2.09 (s, 3H, C(O)C*H_3_*); ^13^C-NMR (100 MHz; CDCl_3_) δ 170.8, 169.7, 169.6, 169.5, 165.7, 165.3, 159.4, 137.9, 137.4, 133.5, 132.5, 130.5, 130.1, 130.0, 129.6, 128.9, 128.8, 128.5, 128.4, 128.3, 128.2, 128.1, 128.0, 127.9, 127.8, 127.5, 113.8, 100.4, 99.4, 99.0, 98.6, 79.9, 78.7, 76.1, 75.8, 75.0, 74.8, 74.4, 74.1, 73.6, 72.6, 69.8, 68.2, 67.1, 63.6, 63.5, 56.3, 55.4, 52.3, 52.0, 20.8; HRMS (FT MS NSI^+^) *m/z* calcd for C_86_H_94_N_7_O_25_ [M+NH_4_]^+^ 1624.6294, found 1624.6299.

*Methyl (6-O-acetyl-2-azido-3-O-benzyl-2-deoxy-4-O-p-methoxybenzyl-α-d-glucopyranosyl-(1→4)-(methyl 2-O-benzoyl-3-O-benzyl-α-l-idopyranosyl) uronate)-(1→4)-6-O-acetyl-2-azido-3-O-benzyl-2-deoxy-α-d-glucopyranosyl-(1→4)-(methyl 2-O-benzoyl-3-O-benzyl-α-l-idopyranoside) uronate* (**6**). Acceptor **4** (167 mg, 0.23 mmol) and donor **1** (286 mg, 0.31 mmol) were mixed together, evaporated from dry toluene (3 × 5 mL) and dried under high vacuum for 1 h. The foam was dissolved in dry DCM (5 mL) and powdered molecular sieves (4 Å, 125 mg) added. The mixture was cooled to 0 °C and NIS (255 mg, 1.14 mmol) added. The mixture was stirred for a further 15 min at this temperature and a catalytic amount (small spatula tip) of AgOTf was then added. The mixture was kept under nitrogen at 0 °C for another 30 min and was then quenched by addition of saturated aqueous Na_2_S_2_O_3_ and saturated aqueous NaHCO_3_ (15 mL, 1:1, *v*/*v*). The suspension was filtered through Celite®, the phases separated and the organic layer washed with saturated aqueous NaCl (10 mL). The organic phase was then dried over MgSO_4_ and solvent removed *in vacuo*. The mixture was purified by column chromatography (DCM/EtOAc 7:1), yielding **6** (270 mg, 0.23 mmol, 77%) as a foam. *R_f_* 0.13 (EtOAc/hexane 1:2); ${\left[\alpha \right]}_{\text{D}}^{20}$ = −1.5 (*c* = 0.31, CH_2_Cl_2_); ^1^H-NMR (400 MHz; CDCl_3_) δ 8.10–8.05 (m, 4H, Bz-Ar*H*), 7.46–7.14 (m, 28H, Ar*H*), 6.84 (d, *J* = 8.7 Hz, 1H, PMB), 5.43 (d, *J* = 4.4 Hz, 1H, H_1’IdoA_), 5.11 (t, *J* = 4.8 Hz, 1H, H_2’IdoA_), 5.03–5.02 (brs, 1H, H_2IdoA_), 5.02–5.01 (brs, 1H, H_1IdoA_), 4.89–4.86 (m, 3H, H_1GlcN_, 2 × C*H*_2_Ph), 4.75 (d, *J* = 2.0 Hz, 1H, H_5IdoA_), 4.73–4.21 (m, 15H, H_1’GlcN_, H_5’IdoA_, H_5’GlcN_, H_6a’GlcN_, H_6aGlcN_, H_3’IdoA_, H_4’IdoA_, 8 × C*H*_2_Ph), 4.09–3.43 (m, 11H, H_3GlcN_, H_3’GlcN_, H_4GlcN_, H_4’GlcN_, H_5GlcN_, H_6b’GlcN_, H_6bGlcN_, H_3IdoA_, H_4IdoA_, 2 × C*H*_2_Ph), 3.77 (s, 3H, C(O)OC*H*_3_), 3.59 (s, 3H, PMBOC*H*_3_), 3.56 (s, 3H, OC*H*_3_), 3.41 (s, 3H, C(O)OC*H*_3_), 3.17 (dd, *J* = 10.2, 3.5 Hz, 1H, H_2GlcN_), 3.13 (dd, *J* = 10.2, 3.6 Hz, 1H, H_2’GlcN_), 2.06 (s, 3H, C(O)C*H*_3_), 1.94 (s, 3H, C(O)C*H*_3_); ^13^C-NMR (100 MHz; CDCl_3_) δ 170.8, 170.6, 169.6, 169.5, 165.6, 165.2, 159.5, 137.7, 137.6, 137.4, 137.2, 133.5, 133.5, 129.9, 128.5, 128.5, 128.4, 128.3, 128.2, 128.1, 127.9, 113.9, 100.4, 99.2, 99.1, 98.4, 80.0, 78.7, 77.4, 77.0, 75.9, 75.6, 75.4, 75.0, 74.7, 74.7, 74.6, 74.0, 72.4, 72.1, 70.4, 70.2, 70.1, 69.6, 68.0, 67.0, 63.6, 63.4, 62.2, 61.8, 56.2, 55.3, 52.3, 52.1, 20.9, 20.8; FT MS NSI^+^ *m/z* calcd for C_81_H_90_N_7_O_26_ [M+NH_4_]^+^ 1576.5930, found 1576.5906.

*Methyl (6-O-acetyl-2-azido-3-O-benzyl-2-deoxy-4-O-p-methoxybenzyl-α-d-glucopyranosyl-(1→4)-(methyl 2-O-benzoyl-3-O-benzyl-α-L-idopyranosyl) uronate)-(1→4)-2-azido-3,6-di-O-benzyl-2-deoxy-α-d-glucopyranosyl-(1→4)-(methyl 2-O-benzoyl-3-O-benzyl-α-l-idopyranosyl) uronate)-(1→4)-6-O-acetyl-2-azido-3-O-benzyl-2-deoxy-α-d-glucopyranosyl-(1→4)-(methyl 2-O-benzoyl-3-O-benzyl-α-l-idopyranoside) uronate* (**7**). Ceric (IV) ammonium nitrate (818 mg, 1.50 mmol) was added to a solution of **5** (1.2 g, 0.75 mmol) in acetonitrile and water (22 mL, 8:1, *v*/*v*). The mixture was stirred for 1 h at ambient temperature, whereupon TLC (EtOAc/hexane, 1:2) showed the reaction to be complete. The solution was diluted with DCM (150 mL), washed with saturated aqueous NaHCO_3_ (100 mL) and saturated aqueous NaCl (50 mL). The organic phase was then dried over MgSO_4_ and solvent removed *in vacuo*. The product was purified by column chromatography (DCM/EtOAc, 30:1), yielding the desired acceptor tetrasaccharide (600 mg, 0.40 mmol, 56%) which was used immediately in the next step. R_f_ = 0.21 (EtOAc/hexane 1:2); HRMS (FT MS NSI^+^) *m/z* calcd for C_78_H_82_N_6_O_24_ [M+NH_4_]^+^ 1504.5719, found 1504.5714. The above acceptor (238 mg, 0.16 mmol) and donor **1** (202 mg, 0.35 mmol) were mixed together, evaporated from dry toluene (3 × 5 mL) and dried under high vacuum for 1 h. The foam was dissolved in dry DCM (4 mL) and powdered molecular sieves (4 Å, 200 mg) added. The mixture was cooled to 0 °C and NIS (180 mg, 0.80 mmol) added. The mixture was stirred for a further 15 min at this temperature and a catalytic amount (small spatula tip) of AgOTf was then added. The mixture was kept under nitrogen at 0 °C for another 30 min and was then quenched by addition of saturated aqueous Na_2_S_2_O_3_ and saturated aqueous NaHCO_3_ (15 mL, 1:1, *v*/*v*). The suspension was filtered through Celite^®^, the phases separated and the organic layer washed with saturated aqueous NaCl (10 mL). The organic phase was then dried over MgSO_4_ and solvent removed *in vacuo*. The crude mixture was purified by column chromatography (EtOAc/hexane, 7:13) to yield **7** (260 mg, 0.11 mmol, 70%). R_f_ = 0.1 (EtOAc/hexane 1:2); ^1^H-NMR (400 MHz; CDCl_3_) δ 8.08–8.02 (m, 4H, Bz-Ar*H*), 7.89–7.86 (m, 2H, Ar*H*), 7.50–6.94 (m, 46H, Ar*H*), 6.81–6.79 (m, 2H, Ar*H*), 5.44 (d, 1H, *J* = 3.9 Hz, H_1IdoA_), 5.40 (d, 1H, *J* = 5.8 Hz, H_1IdoA_), 5.12–5.08 (m, 2H, H_2IdoA_), 4.99 (brs, 1H, H_2IdoA_), 4.95 (brs, 1H, H_1IdoA_), 4.89 (d, 1H, *J* = 3.8 Hz, H_1GlcN_), 4.84–4.82 (m, 2H, H_1GlcN_, C*H*_2_Ar), 4.74–4.66 (m, 7H, H_5IdoA_, 6 × C*H*_2_Ar), 4.63–4.59 (m, 3H, C*H*_2_Ar, 2 × H_5IdoA_), 4.47–4.46 (m, 3H, 2 × C*H*_2_Ar, H_1GlcN_), 4.41 (d, 1H, *J* = 2.5 Hz, H_4IdoA_), 4.38–4.37 (m, 1H, H_4IdoA_), 4.34 (s, 1H, H_4IdoA_), 4.29–3.87 (m, 11H, 6 × H_6GlcN_, 5 × C*H*_2_Ar), 3.84–3.81 (m, 3H, H_3doA_), 3.74 (s, 3H, OC*H*_3_), 3.72–3.66 (m, 4H, C*H*_2_Ar, H_5GlcN_ × 3), 3.62–3.52 (m, 5H, 3 × H_4GlcN_, 2 × H_3GlcN_), 3.48 (s, 3H, OC*H*_3_), 3.42 (s, 3H, OC*H*_3_), 3.38 (s, 3H, OC*H*_3_), 3.37–3.35 (m, 4H, OC*H*_3_, H_3GlcN_), 3.24 (dd, 1H, *J* = 10.3, 3.7 Hz, H_2-GlcN_), 3.15 (dd, 1H, *J* = 10.3, 3.4 Hz, H_2-GlcN_), 3.09 (dd, 1H, *J* = 10.2, 3.6 Hz, H_2-GlcN_), 2.02 (s, 3H, C(O)C*H*_3_), 1.90 (s, 3H, C(O)C*H*_3_); ^13^C-NMR (100 MHz; CDCl_3_) δ 170.8, 170.6, 169.7, 169.5, 169.2, 165.6, 165.3, 165.2, 159.5, 137.8, 137.7, 137.6, 137.4, 137.3, 137.3, 133.6, 133.5, 130.0, 129.9, 129.8, 129.6, 129.3, 129.2, 128.9, 128.8, 128.5, 128.4, 128.2, 128.1, 128.0, 127.9, 127.8 127.6, 127.5, 127.4, 113.9, 100.4, 99.5, 99.2, 98.7, 98.3, 98.0, 80.0, 78.3, 78.2, 77.4, 77.2, 77.0, 76.4, 75.9, 75.8, 75.4, 75.3, 75.2, 75.0, 74.6, 74.4, 74.3, 74.2, 73.7, 73.6, 72.4, 72.3, 71.7, 71.5, 71.2, 70.4, 70.1, 70.0, 69.7, 67.9, 67.5, 67.0, 63.5, 63.4, 63.1, 62.3, 61.7, 56.5, 56.2, 55.4, 52.2, 52.1, 51.7, 29.7, 29.7, 20.9, 20.8; HRMS (FT MS NSI^+^) *m/z* calcd for C_122_H_132_N_11_O_37_ [M+2NH_4_]^2+^ 1173.4522, found 1173.4522.

*Methyl (2-azido-3,6-di-O-benzyl-2-deoxy-4-O-p-methoxybenzyl-α-d-glucopyranosyl-(1→4)-(methyl 2-O-benzoyl-3-O-benzyl-l-idopyranosyl) uronate)-(1→4)-2-azido-3,6-di-O-benzyl-2-deoxy-α-d-gluco-pyranosyl-(1→4)-(methyl 2-O-benzoyl-3-O-benzyl-α-l-idopyranosyl) uronate)-(1→4)-6-O-acetyl-2-azido-3-O-benzyl-2-deoxy-α-d-glucopyranosyl-(1→4)-(methyl 2-O-benzoyl-3-O-benzyl-α-L-ido-pyranoside) uronate* (**8**). See **7** for PMB deprotection of **5**. Acceptor (320 mg, 0.22 mmol) and donor **2** (295 mg, 0.30 mmol) were mixed together, evaporated from dry toluene (3 × 5 mL) and dried under high vacuum for 1 h. The foam was dissolved in dry DCM (6 mL) and powdered molecular sieves (4 Å, 250 mg) added. The mixture was cooled to 0 °C and NIS (240 mg, 1.07 mmol) added. The mixture was stirred for a further 15 min at this temperature and a catalytic amount (small spatula tip) of AgOTf was then added. The mixture was kept under nitrogen at 0 °C for another 30 min and was then quenched by addition of saturated aqueous Na_2_S_2_O_3_ and saturated aqueous NaHCO_3_ (15 mL, 1:1, *v*/*v*). The suspension was filtered through Celite^®^, the phases separated and the organic layer washed with saturated aqueous NaCl (10 mL). The organic phase was then dried over MgSO_4_ and solvent removed *in vacuo*. The crude mixture was purified by column chromatography (EtOAc/hexane, 3:7), to yield **8** (487 mg, 0.21 mmol, 96%). R_f_ = 0.48 (EtOAc/hexane 2:3); ^1^H-NMR (400 MHz; CDCl_3_) δ 8.15–8.09 (m, 4H, Bz-Ar*H*), 7.95–7.93 (m, 2H, Bz-Ar*H*), 7.58–7.27 (m, 33H, Ar*H*), 7.23–7.19 (m, 12H, Ar*H*), 7.10–7.03 (m, 6H, Ar*H*), 6.84–6.82 (m, 2H, PMB), 5.52–5.51 (m, 1H, H_1IdoA_), 5.47 (d, 1H, *J* = 6.0 Hz, H_1IdoA_), 5.20–5.17 (m, 2H, H_2IdoA_), 5.07 (brs, 1H, H_1IdoA_), 5.02 (brs, 1H, H_2IdoA_), 4.98–4.95 (m, 2H, H_1GlcN_), 4.90 (d, 1H, *J* = 11.9 Hz, C*H*_2_Ph), 4.81–4.20 (m, 24H, H_1GlcN_, 3 ×H_5IdoA_, H_3IdoA_, 6 × H_6GlcN_, 11 × C*H*_2_Ph), 4.08–4.01 (m, 4H, 3 × H_4IdoA_, C*H*_2_Ph), 3.96–3.55 (m, 18H, 5 × C*H*_2_Ph, H_3GlcN_, 3 × H_4GlcN_, 3 × H_5GlcN_, C(O)OC*H*_3_, PMBOC*H*_3_), 3.46–3.37 (m, 11H, 2 × C(O)OC*H*_3_, OC*H*_3_, 2 × H_3GlcN_), 3.33–3.29 (m, 1H, H_2GlcN_), 3.27–3.23 (m, 1H, H_2GlcN_), 3.16 (dd, 1H, *J* = 10.2, 3.6 Hz, H_2GlcN_), 2.10–2.07 (s, 3H, C(O)C*H*_3_); ^13^C-NMR (100 MHz; CDCl_3_) δ 170.7, 169.7, 169.5, 169.2, 165.6, 165.2, 137.9, 137.8, 137.7, 137.4, 137.3, 137.2, 133.5, 130.0, 129.9, 129.8, 129.5, 129.4, 129.3, 129.2, 128.8, 128.7, 128.4, 128.4, 128.3, 128.1, 128.0, 128.0, 127.9, 127.8, 127.7, 127.6, 127.4, 127.3, 113.7, 100.3, 99.6, 99.1, 98.7, 98.2, 98.0, 79.8, 78.3, 75.9, 75.7, 75.2, 74.9, 74.6, 74.5, 74.4, 74.3, 74.2, 73.6, 73.5, 72.4, 71.8, 71.7, 71.4, 71.3, 70.7, 69.7, 67.9, 67.7, 67.1, 63.4, 63.3, 61.6, 56.2, 55.3, 52.1, 51.9, 51.7, 20.9; HRMS (FT MS NSI^+^) *m/z* calcd for C_127_H_139_N_11_O_36_ [M+2NH_4_]^2+^ 1197.4704, found 1197.4706.

*Methyl (6-O-acetyl-2-azido-3-O-benzyl-2-deoxy-4-O-p-methoxy-m-iodobenzyl-α-d-glucopyranosyl-(1→4)-(methyl 2-O-benzoyl-3-O-benzyl-α-l-idopyranosyl) uronate)-(1→4)-2-azido-3,6-di-O-benzyl-2-deoxy-α-d-glucopyranosyl-(1→4)-(methyl 2-O-benzoyl-3-O-benzyl-α-l-idopyranosyl) uronate)-(1→4)-2-azido-3,6-di-O-benzyl-2-deoxy-α-d-glucopyranosyl-(1→4)-(methyl 2-O-benzoyl-3-O-benzyl-α-l-idopyranosyl) uronate)-(1→4)-6-O-acetyl-2-azido-3-O-benzyl-2-deoxy-α-d-glucopyranosyl-(1→4)-(methyl 2-O-benzoyl-3-O-benzyl-α-l-idopyranoside) uronate* (**9**). Ceric (IV) ammonium nitrate (210 mg, 0.38 mmol) was added to a solution of **8** (450 mg, 0.19 mmol) in acetonitrile and water (4 mL, 8:1, *v*/*v*). The mixture was stirred for 1 h at ambient temperature, whereupon TLC (EtOAc/hexane, 2:3) showed the reaction to be complete. The solution was diluted with DCM (100 mL), washed with saturated aqueous NaHCO_3_ (50 mL) and saturated aqueous NaCl (50 mL). The organic phase was then dried over MgSO_4_ and solvent removed *in vacuo*. The product was purified by column chromatography (DCM/EtOAc, 12:1), yielding the desired acceptor hexasaccharide (330 mg, 0.35 mmol, 78%) which was used immediately in the next step. R_f_ = 0.38 (EtOAc/hexane, 2:3); HRMS (FT MS NSI^+^) *m/z* calcd for C_119_H_131_N_11_O_35_ [M+2NH_4_]^2+^ 1137.4416, found 1137.4416. The above acceptor (294 mg, 0.13 mmol) and donor **1** (166 mg, 0.18 mmol) were mixed together, evaporated from dry toluene (3 × 5 mL) and dried under high vacuum for 1 h. The foam was dissolved in dry DCM (4 mL) and powdered molecular sieves (4 Å, 200 mg) added. The mixture was cooled to 0 °C and NIS (200 mg, 0.89 mmol) added. The mixture was stirred for a further 15 min at this temperature and a catalytic amount (small spatula tip) of AgOTf was then added. The mixture was kept under nitrogen at 0 °C for another 30 min and was then quenched by addition of saturated aqueous Na_2_S_2_O_3_ and saturated aqueous NaHCO_3_ (15 mL, 1:1, *v*/*v*). The suspension was filtered through Celite^®^, the phases separated and the organic layer washed with saturated aqueous NaCl (10 mL). The organic phase was then dried over MgSO_4_ and solvent removed *in vacuo*. The crude product was purified by column chromatography (EtOAc/hexane, 2:7), to yield **9** (265 mg, 0.09 mmol, 66%). R_f_ = 0.40 (EtOAc/hexane 2:3); ^1^H-NMR (400 MHz; CDCl_3_) δ 8.07 (d, *J* = 7.2 Hz, 2H, Ar*H*), 8.02 (d, *J* = 6.6 Hz, 2H, Ar*H*), 7.89 (d, *J* = 7.3 Hz, 2H, Ar*H*), 7.84 (d, *J* = 7.3 Hz, 2H, Ar*H*), 7.56 (d, *J* = 2.0 Hz, 1H, Ar*H*), 7.52–6.88 (m, 68H, Ar*H*), 6.68 (d, *J* = 8.5 Hz, 1H, Ar*H*), 5.47 (d, *J* = 5.1 Hz, 1H, H_1IdoA_), 5.43 (d, *J* = 3.5 Hz, 1H, H_1IdoA_), 5.38 (d, *J* = 5.9 Hz, 1H, H_1IdoA_), 5.12–5.07 (m, 3H, H_2IdoA_), 4.98 (s, 1H, H_2IdoA_), 4.94 (s, 1H, H_1IdoA_), 4.88–4.80 (m, 4H, 3 × H_1GlcN_, C*H*_2_Ar), 4.73–4.58 (m, 12H, 11 × C*H*_2_Ar, H_5IdoA_), 4.51–4.11 (m, 18H, H_1GlcN_, 4 × H_4IdoA_, 3 × H_5IdoA_, 10 × C*H*_2_Ar), 3.99–3.93 (m, 6H, 2 × H_6-GlcN_ 4 × H_3IdoA_), 3.86–3.66 (m, 9H, 6 × H_6-GlcN_, OC*H*_3_), 3.56–3.21 (m, 29H, 4 × H_5-GlcN_, 4 × H_3-GlcN_, 4 × H_4-GlcN_, 5 × OC*H*_3_, 2 × H_2-GlcN_), 3.14 (dd, *J* = 10.2, 3.4 Hz, 1H, H_2-GlcN_), 3.07 (dd, *J* = 10.2, 3.5 Hz, 1H, H_2-GlcN_), 2.01 (s, 3H, C(O)C*H*_3_), 1.90 (s, 3H, C(O)C*H*_3_); ^13^C-NMR (100 MHz; CDCl_3_) δ 177.3, 170.8, 170.5, 169.7, 169.5, 169.3, 169.2, 165.5, 165.2, 165.2, 157.9, 139.3, 137.8, 137.8, 137.7, 137.6, 137.5, 137.3, 133.6, 133.5, 131.9, 130.0, 129.9, 129.9, 129.8, 129.7, 129.6, 129.5, 129.3, 129.2, 129.2, 128.8, 128.7, 128.5, 128.5, 128.4, 128.4, 128.4, 128.3, 128.2, 128.2, 128.1, 128.1, 128.0, 127.9, 127.9, 127.9, 127.8, 127.8, 127.6, 127.5, 127.5, 127.4, 127.3, 127.2, 110.7, 100.3, 99.5, 99.1, 98.7, 98.3, 98.0, 97.9, 80.0, 78.2, 78.02, 77.4, 77.0, 76.5, 75.9, 75.9, 75.7, 75.4, 75.2, 75.0, 74.9, 74.6, 74.5, 74.4, 74.2, 73.7, 73.6, 73.6, 72.4, 72.2, 71.8, 71.4, 70.6, 70.0, 69.7, 69.4, 67.8, 67.5, 67.3, 66.9, 63.5, 63.3, 63.2, 62.8, 62.1, 61.6, 56.4, 56.3, 56.2, 55.3, 52.2, 52.1, 51.7, 51.6, 29.8, 29.6; HRMS (FT MS NSI^+^) *m/z* calcd for C_163_H_175_N_14_O_48_I [M+2NH_4_]^2+^ 1,612.0375, found 1,612.0377.

## 4. Conclusions

A modular synthetic access to differentially protected H/HS-like oligosaccharides is demonstrated using two d-GlcN-l-IdoA modules with differentiated d-GlcN *O*-6 protecting groups, suitable for ultimate installation of either a 6-OH or 6-OS moiety. This is applied to generate a ladder of tetrasaccharide, hexasaccharide and octasaccharide systems which retain a common reducing end 6-OAc, either as the only acylated *O*-6, or combined with double-terminal units to provide oligosaccharides withterminal d-GlcN units both bearing *O*-6 acylations. High stereochemical integrity in synthesis is evidenced by NMR spectra, which allow ready comparisons of differentiated l-IdoA H-1 across the series. This approach should facilitate wider access to medium length heparin-like oligosaccharides with ready programming of site-specific changes at *O*-6 sites along different backbones.

## Supplementary Materials

Supplementary materials (1D and 2D NMR). Supplementary materials can be accessed at: http://www.mdpi.com/1420-3049/20/04/6167/s1.
