# Supplementary material for: Modular Synthesis of Heparin-Related Tetra-, Hexa- and Octasaccharides with Differential O-6 Protections: Programming for Regiodefined 6-O-Modifications
Source: Molecules. 2015 Apr 9;20(4):6167–80. doi: 10.3390/molecules20046167 (PMC4421873; doi:10.3390/molecules20046167)

Supplementary File

Methyl 6-*O*-acetyl-2-azido-3-*O*-benzyl-2-deoxy-α-D-glucopyranosyl-(1→4)-(methyl 2-*O*-benzoyl-3-*O*-benzyl-α-L-idopyranoside) uronate (4)

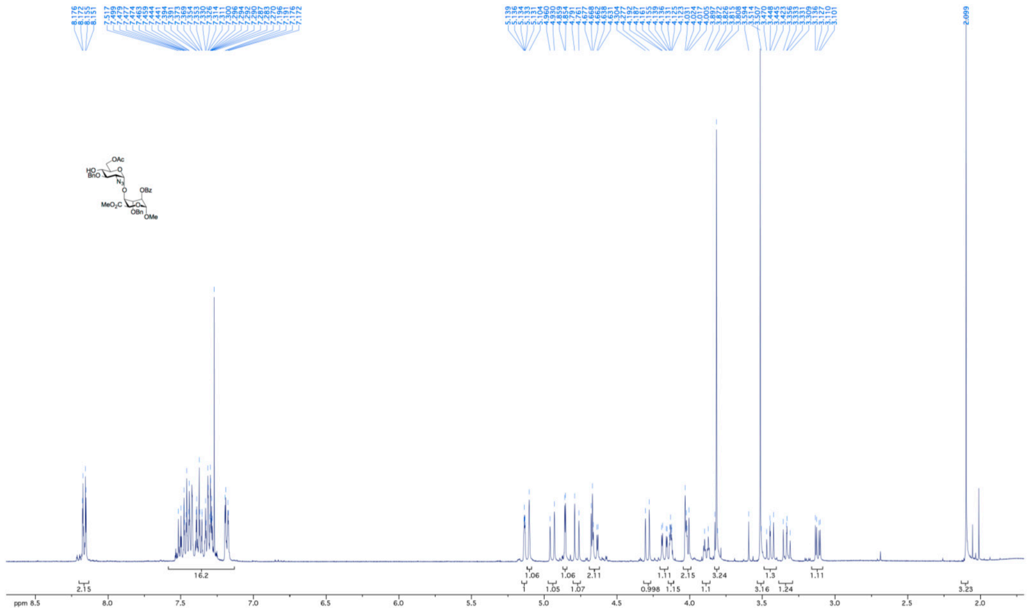

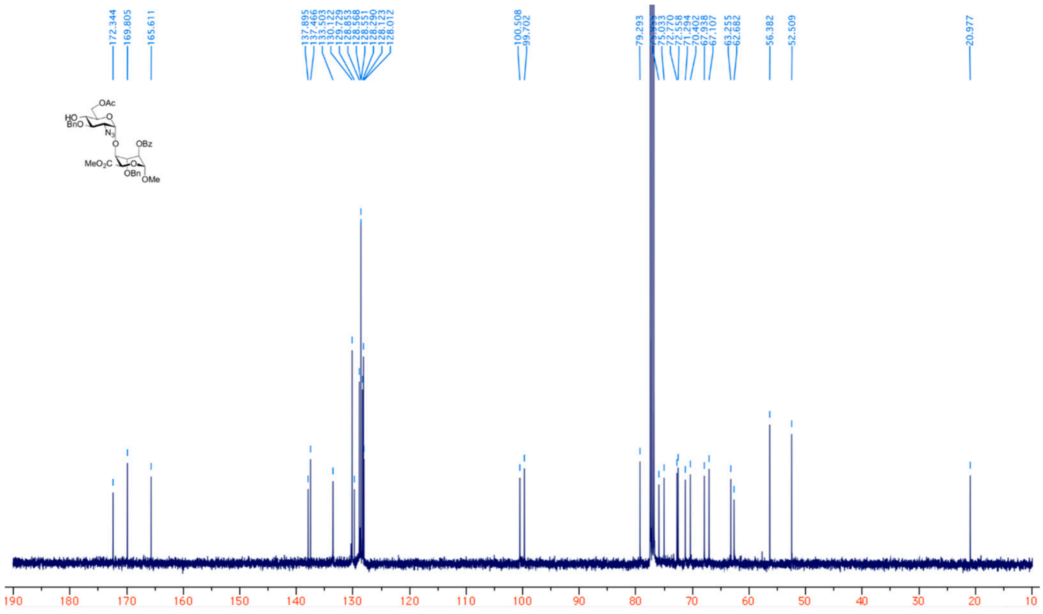

Methyl (2-azido-3,6-di-*O*-benzyl-2-deoxy-4-*O*-p-methoxybenzyl-α-D-glucopyranosyl-(1-4)-(methyl 2-*O*-benzoyl-3-*O*-benzyl-α-L-idopyranosyl) uronate)-(1-4)-6-*O*-acetyl-2-azido-3-*O*-benzyl-2-deoxy-α-D-glucopyranosyl-(1-4)-(methyl 2-*O*-benzoyl-3-*O*-benzyl-α-L-idopyranoside) uronate (5)

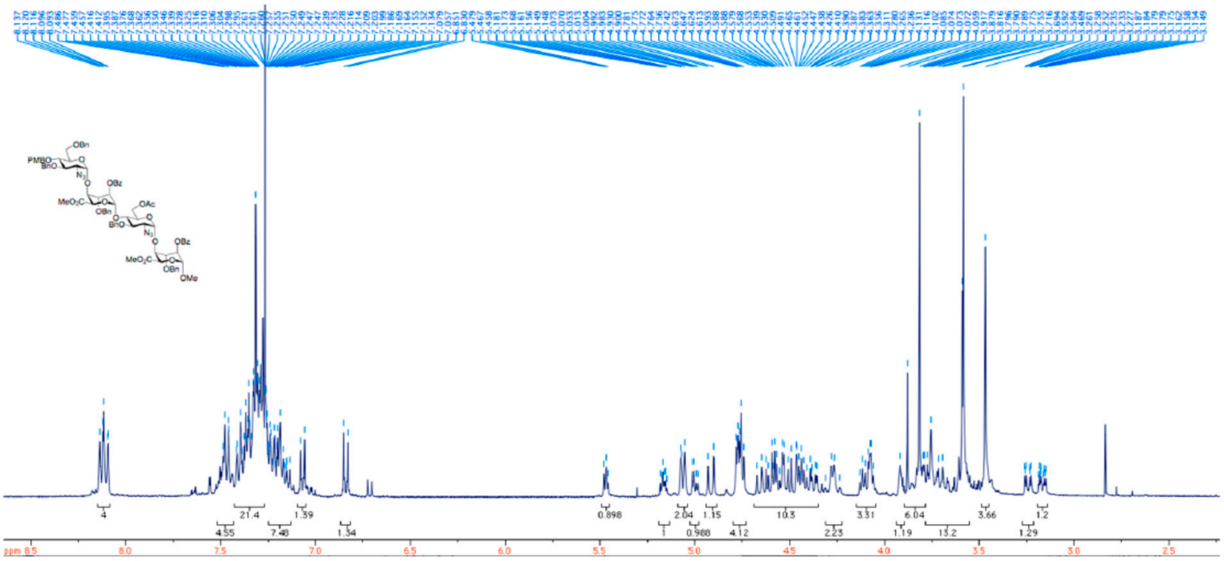

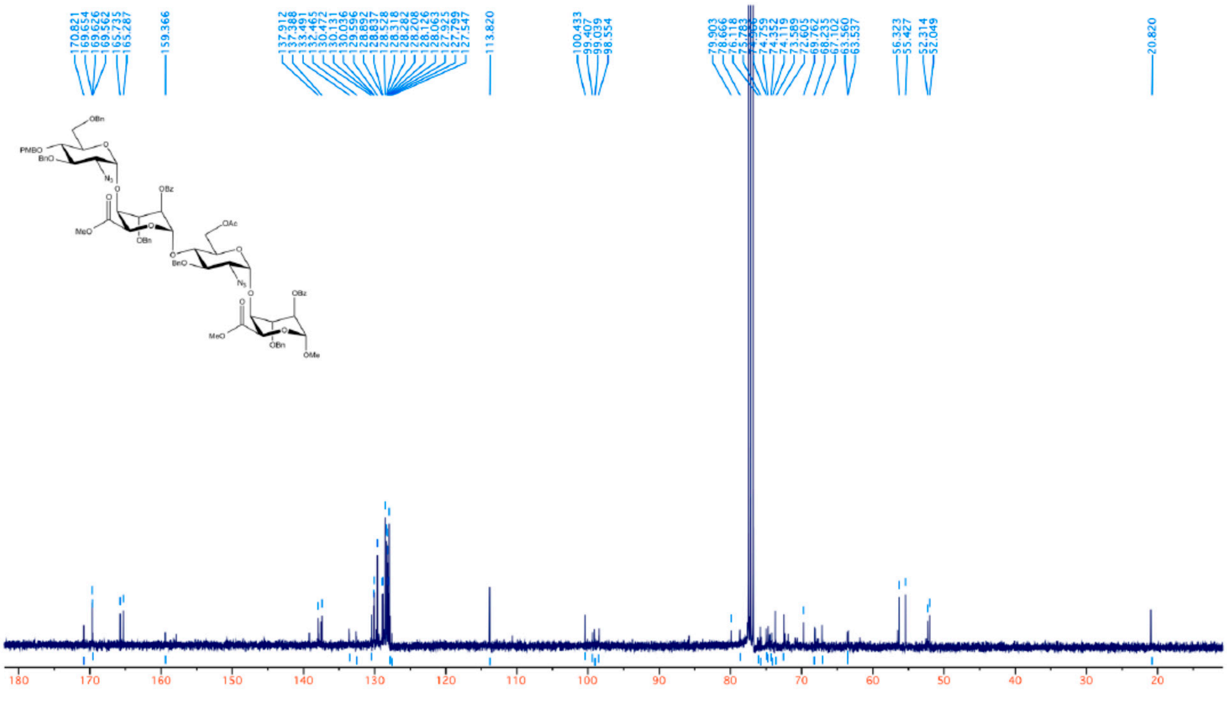

Methyl (2-azido-3,6-di-*O*-benzyl-2-deoxy-4-hydroxy-α-D-glucopyranosyl-(1-4)-(methyl 2-*O*-benzoyl-3-*O*-benzyl-α-L-idopyranosyl) uronate)-(1-4)-6-*O*-acetyl-2-azido-3-Obenzyl-2-deoxy-α-D-glucopyranosyl-(1-4)-(methyl 2-*O*-benzoyl-3-*O*-benzyl-α-Lidopyranoside)uronate (O4 deprotection of 5). LCMS Data 1.

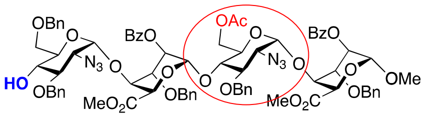

Display Report

Analysis Info

Analysis Name	MB 05-5 GMA4.D	Acquisition Date	13/02/2015 16:22:49
Method	90ACN.M	Operator	mib
Sample Name	MB 05-5/GMA4	Instrument	LC-MSD-Trap-SL
Comment	MB 05-5/GMA4<br>Kinetex XB C18 4.6x100mm 5u<br>90ACN 1ml/mn +ve APCI		

Acquisition Parameter

Ion Source Type	APCI	Ion Polarity	Positive	Alternating Ion Polarity	off
Mass Range Mode	Std/Normal	Scan Begin	500 m/z	Scan End	2200 m/z
Capillary Exit	181.0 Volt	Skim 1	40.0 Volt	Trap Drive	131.0
Accumulation Time	200000 µs	Averages	7 Spectra	Auto MS/MS	off

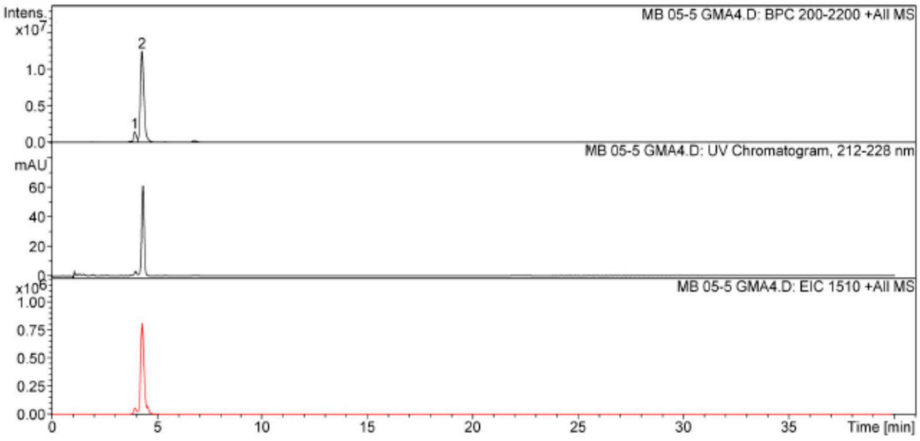

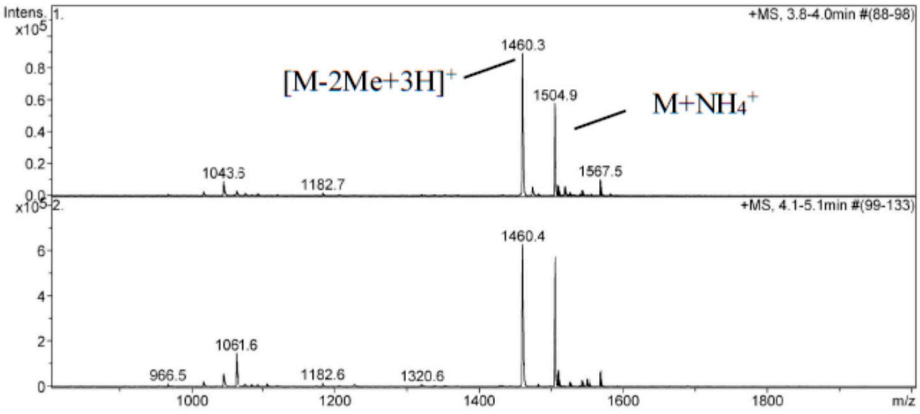

Data File C:\HPCHEM\1\DATA\CD 90ACN 13FEB15\MB 05-5 GMA4.D
Sample Name: MB 05-5/GMA4

===

Acq. Operator : Rehana   Seq. Line : 2
Acq. Instrument : Instrument 1   Location : Vial 4
Injection Date : 13/02/2015 16:22:31   Inj : 1
                                          Inj Volume : 50 µl
Different Inj Volume from Sequence !   Actual Inj Volume : 10 µl
Acq. Method : C:\HPCHEM\1\METHODS\Rehana APCI LCMS\90ACN.M
Last changed : 13/02/2015 16:21:29 by Rehana
Analysis Method : C:\HPCHEM\1\METHODS\Rehana APCI LCMS\REHANA 90ACN APCI LCMS.M
Last changed : 16/02/2015 10:14:50 by Rehana
Sample Info : MB 05-5/GMA4
Kinetex XB C18 4.6x100mm 5u
90ACN 1ml/min +ve APCI

===

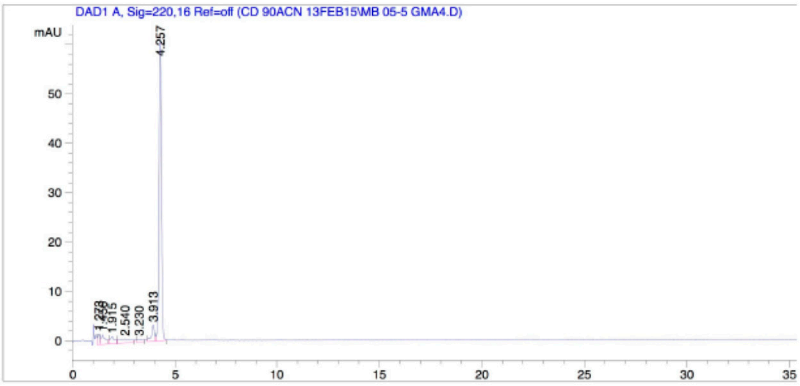

===

Area Percent Report

===

Sorted By : Signal
Multiplier : 1.0000
Dilution : 1.0000
Use Multiplier & Dilution Factor with ISTDs

Signal 1: DAD1 A, Sig=220,16 Ref=off

Peak #	RetTime [min]	Type	Width [min]	Area [mAU*s]	Height [mAU]	Area %
1	1.273	VV	0.1066	16.18228	2.13032	2.6040
2	1.456	VB	0.1648	24.76468	1.91614	3.9851
3	1.915	BB	0.1833	21.19610	1.47791	3.4109
4	2.540	BB	0.4499	29.61157	8.03640e-1	4.7651
5	3.230	BB	0.2443	10.34769	5.58001e-1	1.6651
6	3.913	BV	0.1470	33.44304	3.27741	5.3816
7	4.257	VB	0.1232	485.88519	60.85077	78.1882

Totals : 621.43057 71.01421

===

*** End of Report ***

Methyl (6-*O*-acetyl-2-azido-3-*O*-benzyl-2-deoxy-4-*O*-p-methoxybenzyl-α-Dglucopyranosyl-(1-4)-(methyl 2-*O*-benzoyl-3-*O*-benzyl-α-L-idopyranosyl) uronate)-(1-4)-6-*O*-acetyl-2-azido-3-*O*-benzyl-2-deoxy-α-D-glucopyranosyl-(1-4)-(methyl 2-Obenzoyl-3-*O*-benzyl-α-L-idopyranoside) uronate (6)

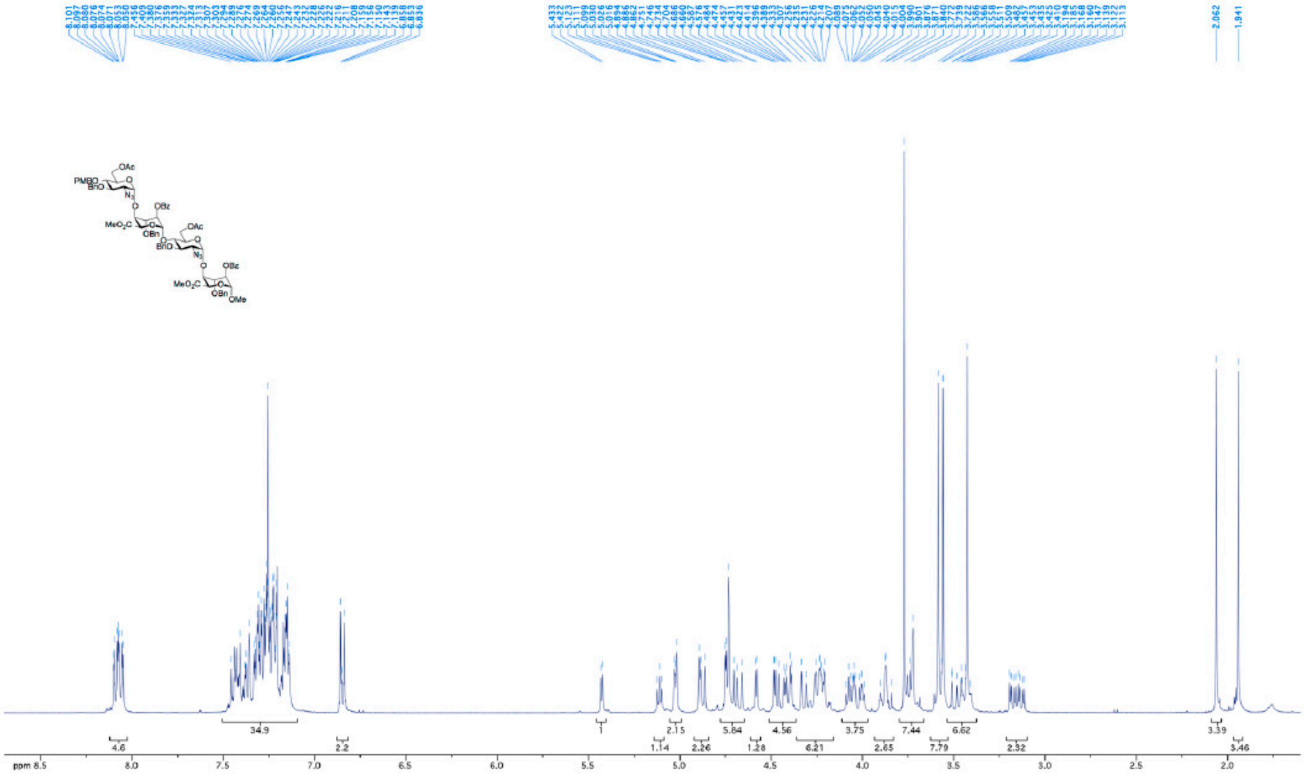

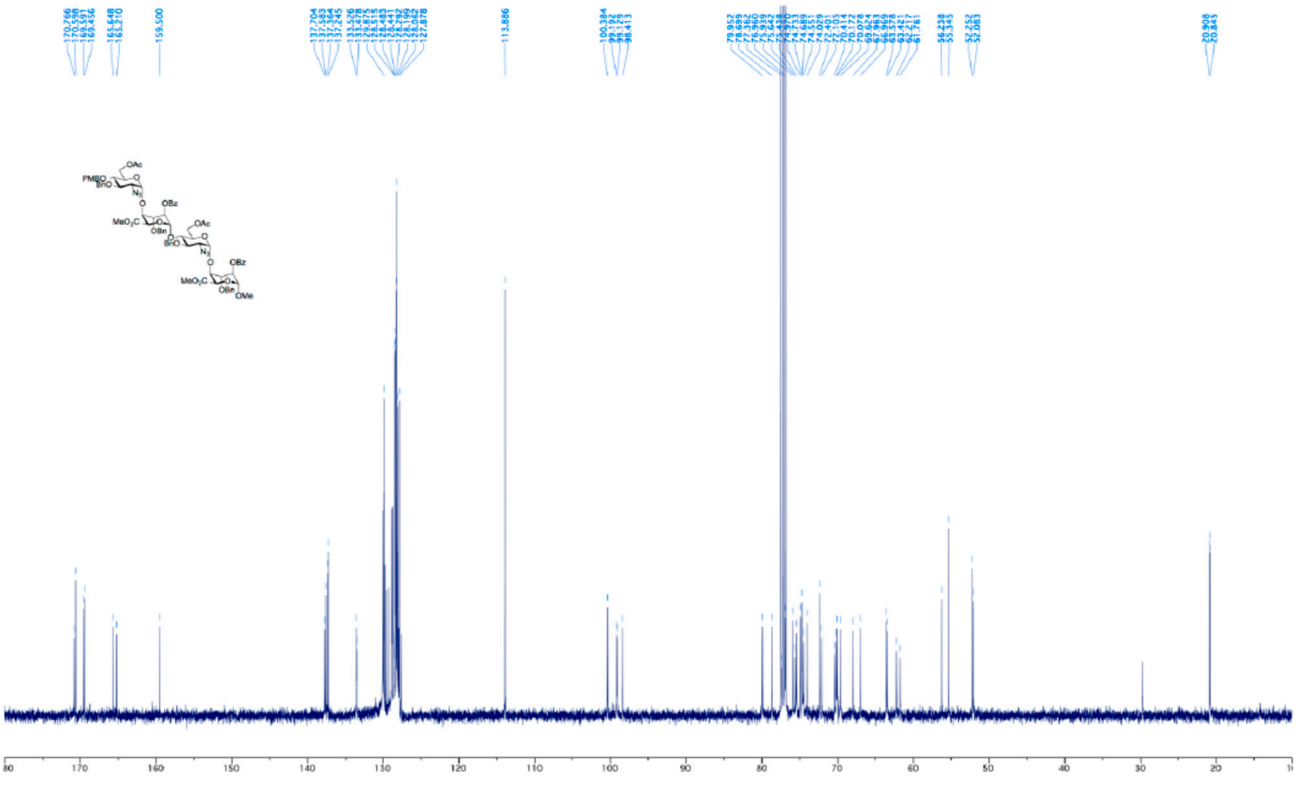

Methyl (6-*O*-acetyl-2-azido-3-*O*-benzyl-2-deoxy-4-*O*-p-methoxybenzyl-α-Dglucopyranosyl-(1-4)-(methyl 2-*O*-benzoyl-3-*O*-benzyl-α-L-idopyranosyl) uronate)-(1-4)-2-azido-3,6-di-*O*-benzyl-2-deoxy-α-D-glucopyranosyl-(1-4)-(methyl 2-*O*-benzoyl-3-*O*-benzyl-α-L-idopyranosyl) uronate)-(1-4)-6-*O*-acetyl-2-azido-3-*O*-benzyl-2-deoxy-α-D-glucopyranosyl-(1-4)-(methyl 2-*O*-benzoyl-3-*O*-benzyl-α-L-idopyranoside) urinate (7)

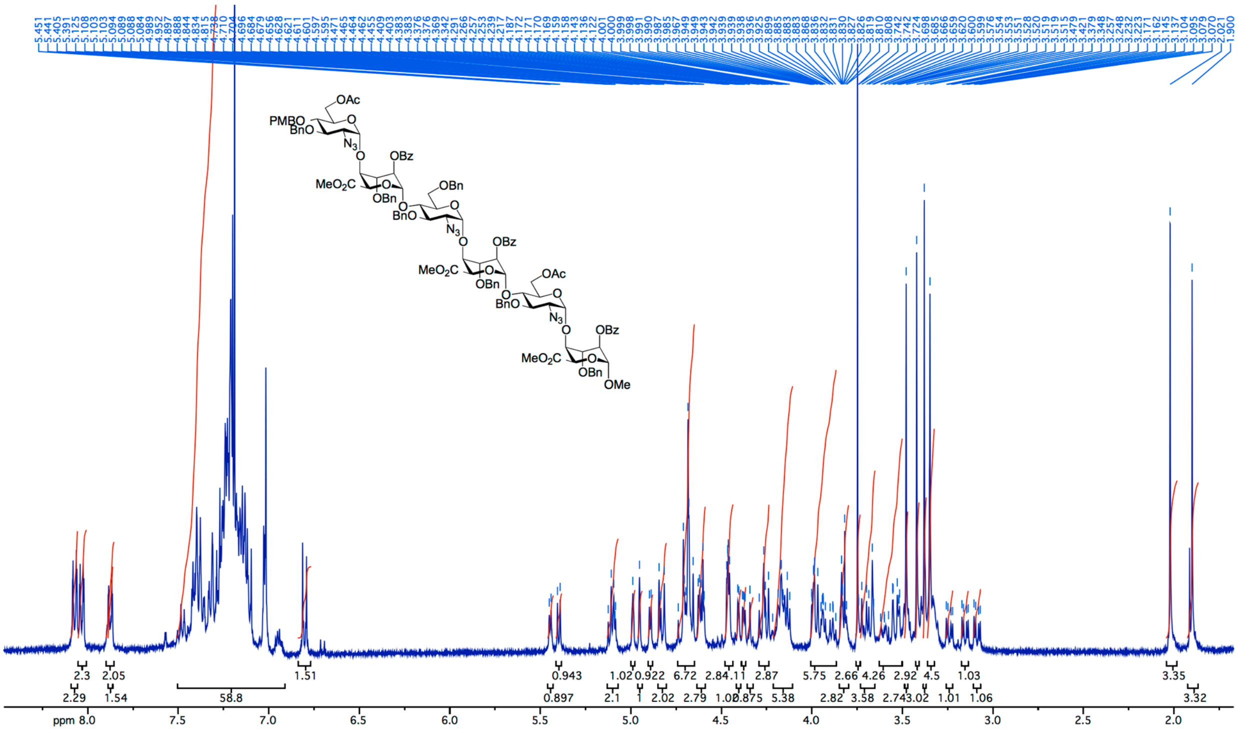

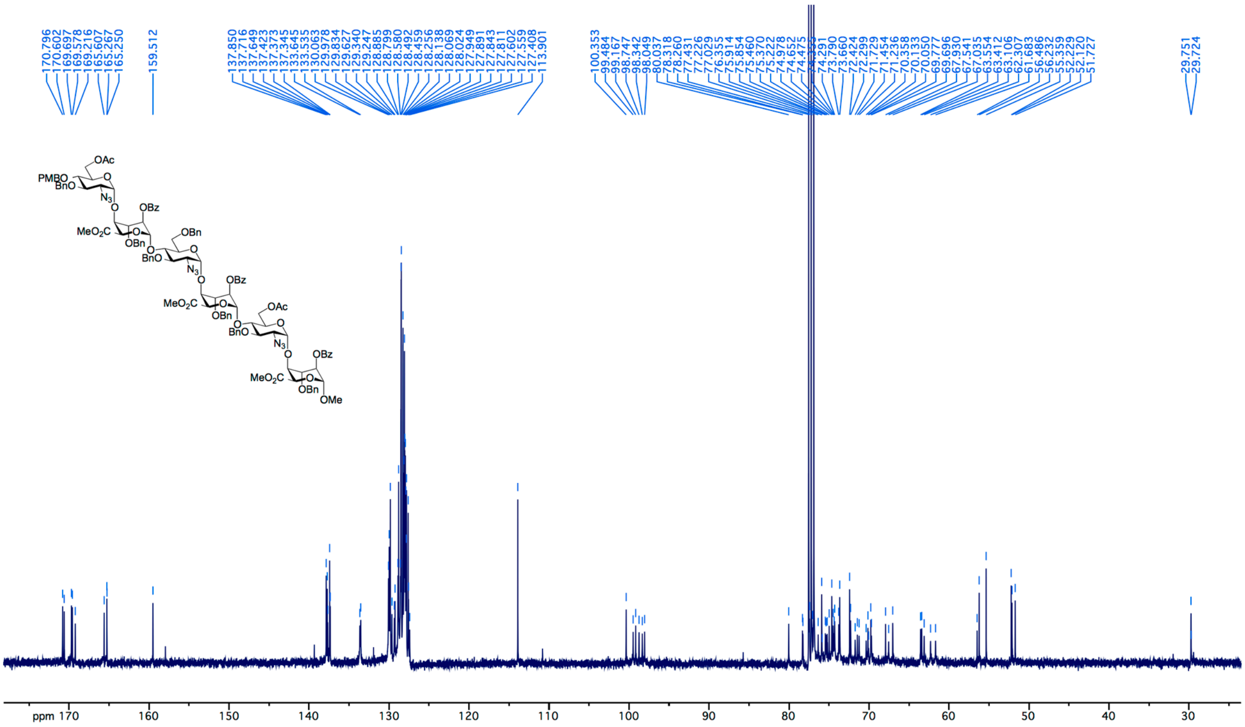

Methyl (6-*O*-acetyl-2-azido-3-*O*-benzyl-2-deoxy-4-*O*-p-methoxybenzyl-α-Dglucopyranosyl-(1-4)-(methyl 2-*O*-benzoyl-3-*O*-benzyl-α-L-idopyranosyl) uronate)-(1-4)-2-azido-3,6-di-*O*-benzyl-2-deoxy-α-D-glucopyranosyl-(1-4)-(methyl 2-*O*-benzoyl-3-*O*-benzyl-α-L-idopyranosyl) uronate)-(1-4)-6-*O*-acetyl-2-azido-3-*O*-benzyl-2-deoxy-α-D-glucopyranosyl-(1-4)-(methyl 2-*O*-benzoyl-3-*O*-benzyl-α-L-idopyranoside) urinate (7)

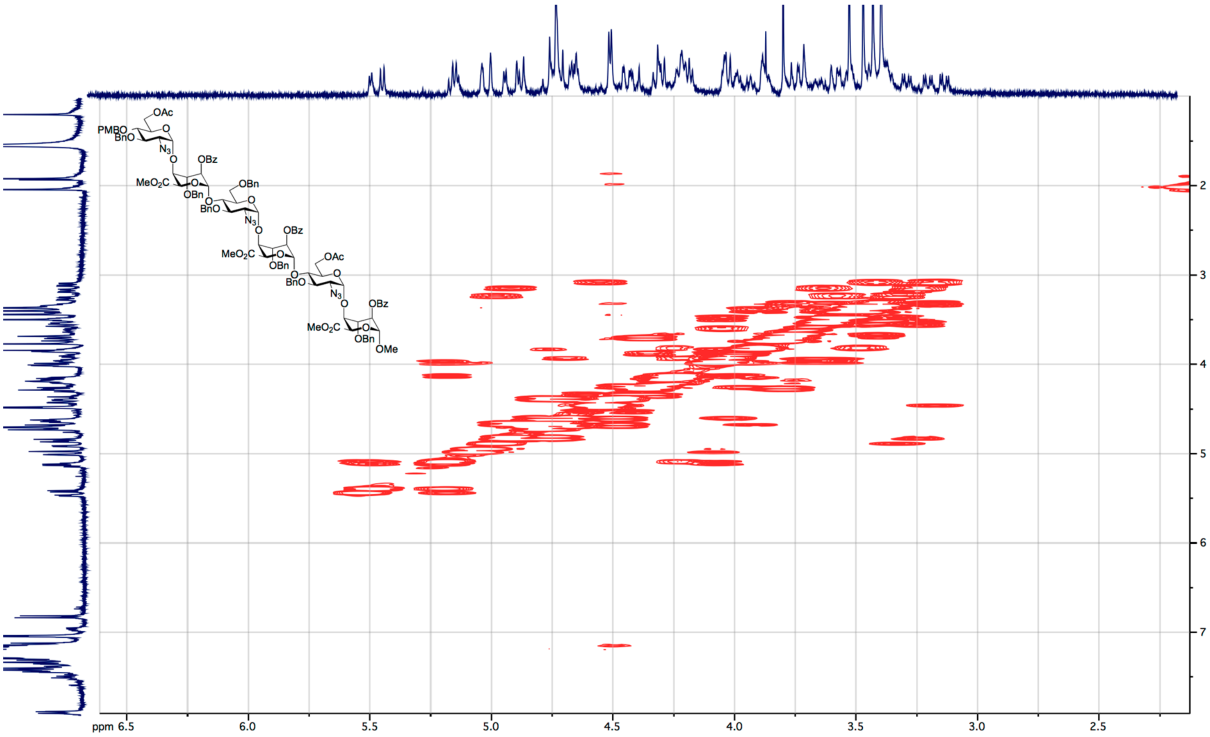

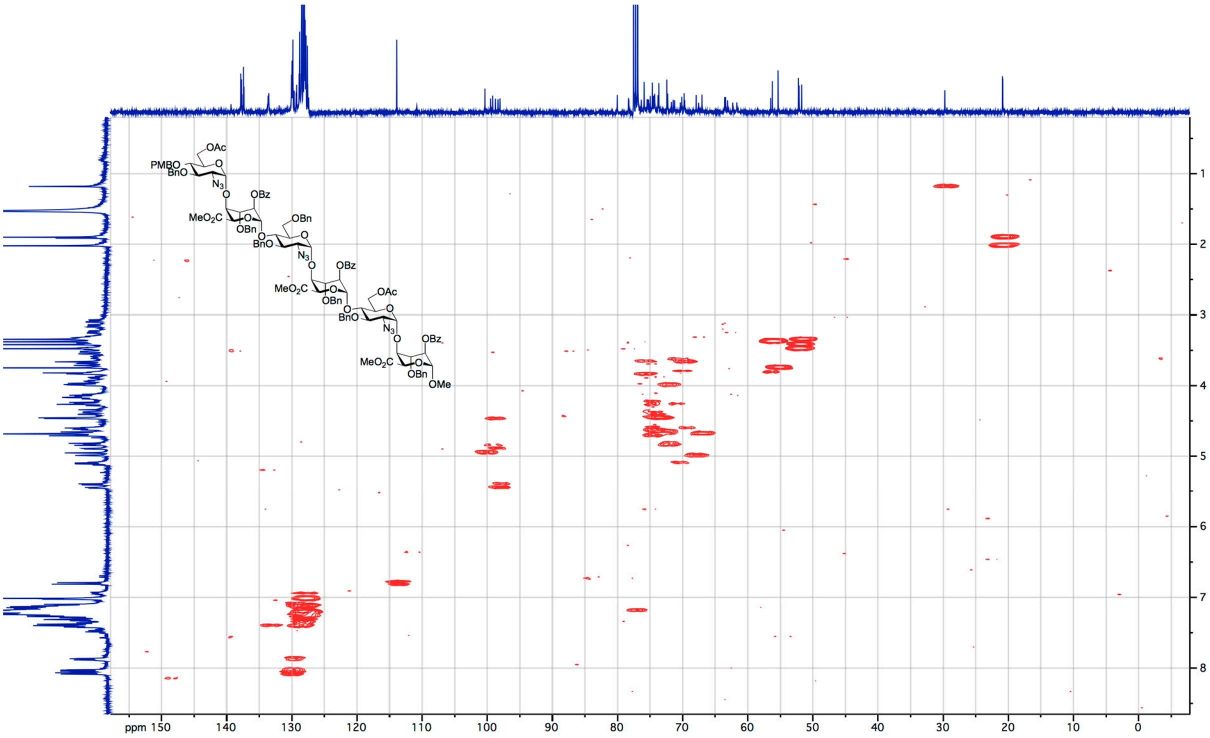

Methyl (2-azido-3,6-di-*O*-benzyl-2-deoxy-4-*O*-*p*-methoxybenzyl-α-D-glucopyranosyl-(1→4)-(methyl 2-*O*-benzoyl-3-*O*-benzyl-α-L-idopyranosyl) uronate)-(1→4)-2-azido-3,6-di-*O*-benzyl-2-deoxy-α-D-glucopyranosyl-(1→4)-(methyl 2-*O*-benzoyl-3-*O*-benzyl-α-L-idopyranosyl) uronate)-(1→4)-6-*O*-acetyl-2-azido-3-*O*-benzyl-2-deoxy-α-D-glucopyranosyl-(1→4)-(methyl 2-*O*-benzoyl-3-*O*-benzyl-α-L-idopyranoside) uronate (8)

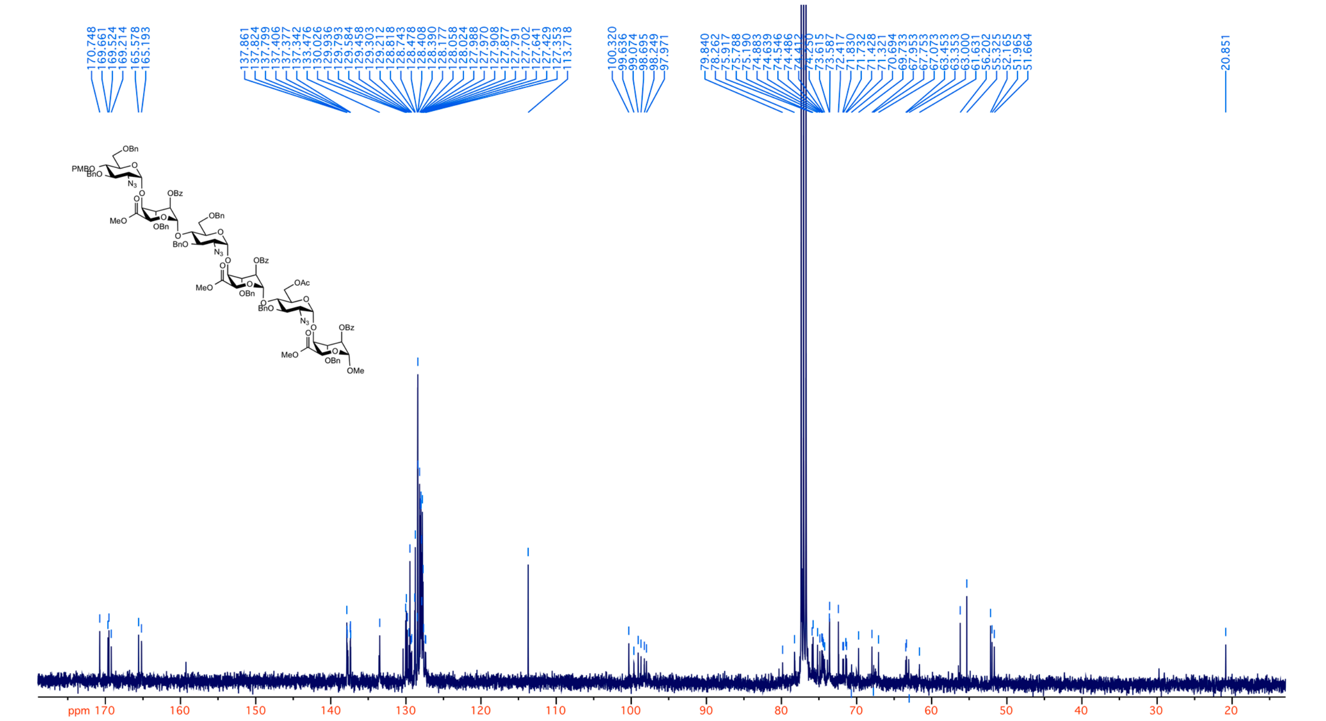

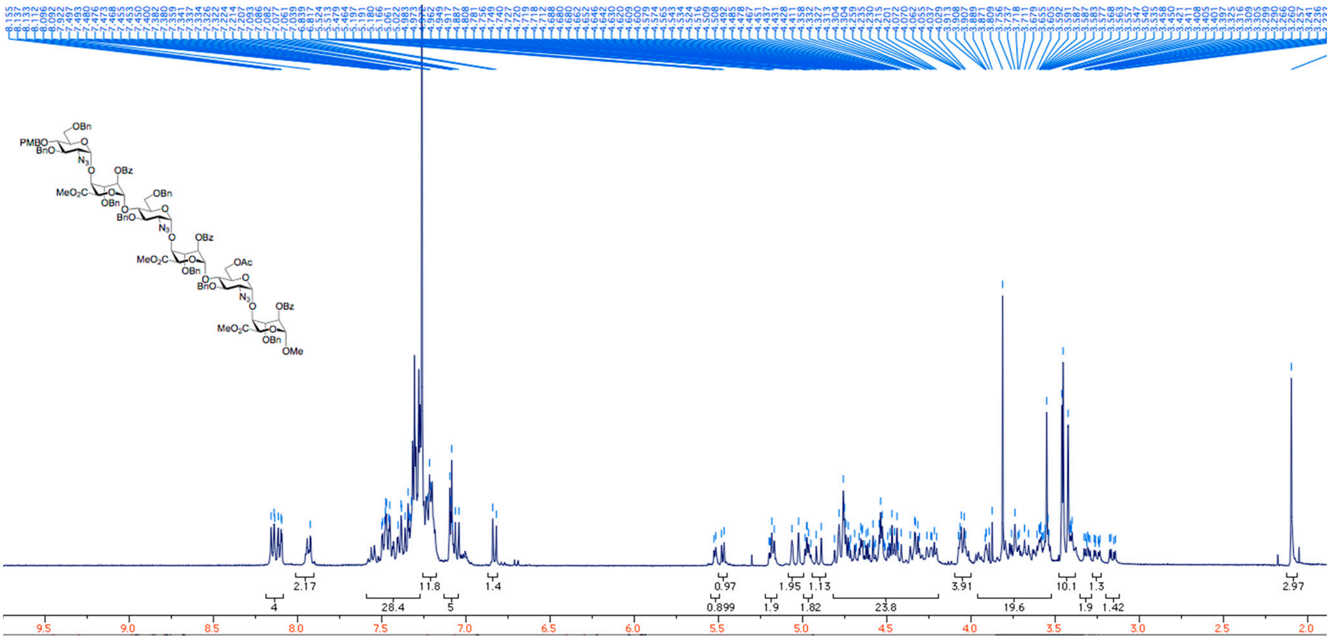

Methyl (2-azido-3,6-di-*O*-benzyl-2-deoxy-4-hydroxy-α-D-glucopyranosyl-(1→4)-(methyl 2-*O*-benzoyl-3-*O*-benzyl-α-L-idopyranosyl) uronate)-(1→4)-2-azido-3,6-di-*O*-benzyl-2-deoxy-α-D-glucopyranosyl-(1→4)-(methyl 2-*O*-benzoyl-3-*O*-benzyl-α-L-idopyranosyl)uronate)-(1→4)-6-*O*-acetyl-2-azido-3-*O*-benzyl-2-deoxy-α- D-glucopyranosyl-(1→4)-(methyl 2-*O*-benzoyl-3-*O*-benzyl-α-L-idopyranoside) uronate (O4 deprotection of 8). LCMS 1.

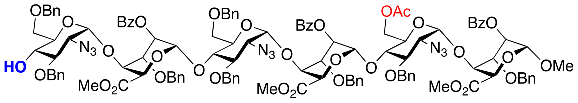

Display Report

Analysis Info

Analysis Name	MB 05-11 GMA6.D	Acquisition Date	16/02/2015 11:38:04
Method	100ACN.M	Operator	mib
Sample Name	MB 05-11/GMA6	Instrument	LC-MSD-Trap-SL
Comment	MB 05-11/GMA6<br>Kinetex XB C18 4.6x100mm 5u<br>100ACN 1ml/min +ve APCI		

Acquisition Parameter

Ion Source Type	APCI	Ion Polarity	Positive	Alternating Ion Polarity	off
Mass Range Mode	Extended	Scan Begin	500 m/z	Scan End	3000 m/z
Capillary Exit	211.0 Volt	Skim 1	40.0 Volt	Trap Drive	170.7
Accumulation Time	200000 μs	Averages	7 Spectra	Auto MS/MS	off

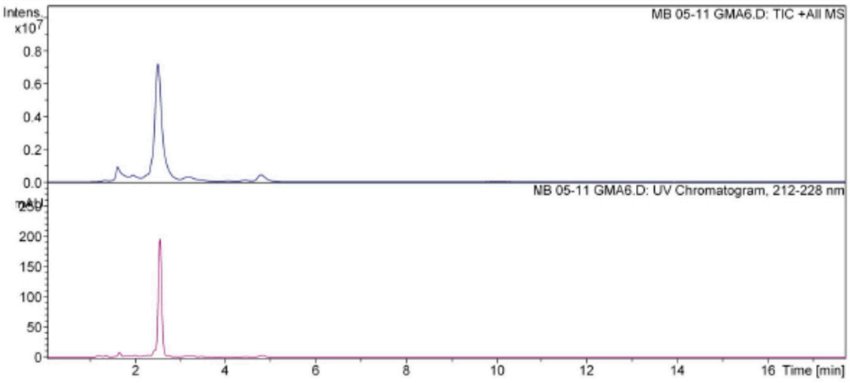

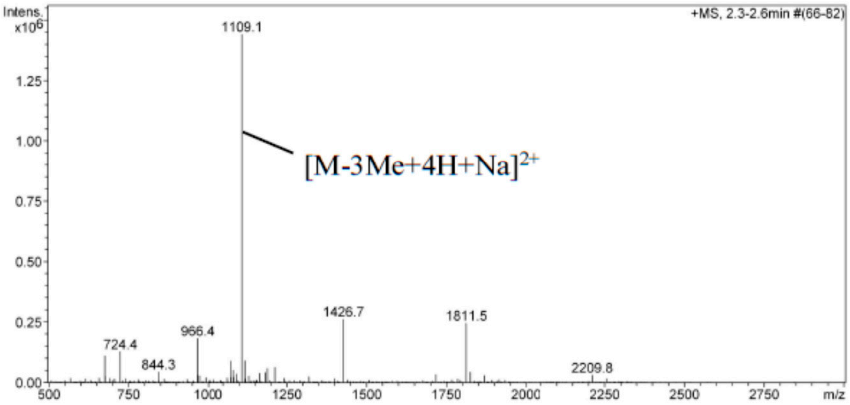

```
Data File C:\HPCHEM\1\DATA\CD 100ACN 16FEB15\MB 05-11 GMA6.D
 Sample Name: MB 05-11/GMA6
    ======================================================================
    Acq. Operator   : Rehana                          Seq. Line :   2
    Acq. Instrument : Instrument 1                     Location : Vial 6
    Injection Date  : 16/02/2015 11:37:39                    Inj :   1
                                                    Inj Volume : 50 µl
    Different Inj Volume from Sequence !     Actual Inj Volume : 10 µl
    Acq. Method      : C:\HPCHEM\1\METHODS\Rehana APCI LCMS\100ACN.M
    Last changed     : 16/02/2015 11:36:43  by Rehana
    Analysis Method : C:\HPCHEM\1\METHODS\Rehana APCI LCMS\REHANA 90ACN APCI LCMS.M
    Last changed     : 16/02/2015 10:14:50  by Rehana
    Sample Info      : MB 05-11/GMA6
                       Kinetex XB C18 4.6x100mm 5u
                       100ACN 1ml/min +ve APCI

    ======================================================================
```

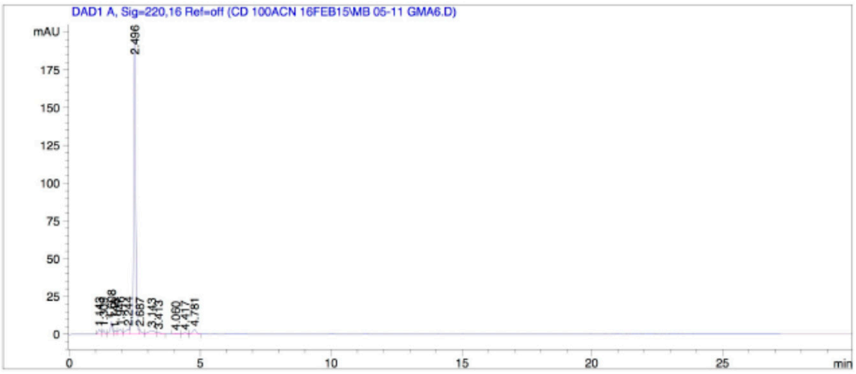


```
    ======================================================================
                         Area Percent Report
    ======================================================================

    Sorted By             :      Signal
    Multiplier            :      1.0000
    Dilution              :      1.0000
    Use Multiplier & Dilution Factor with ISTDs


    Signal 1: DAD1 A, Sig=220,16 Ref=off
```

Peak #	RetTime [min]	Type	Width [min]	Area [mAU*s]	Height [mAU]	Area %
1	1.143	BV	0.0941	21.93324	3.19534	1.6290
2	1.309	VV	0.0721	14.80308	2.97028	1.0994
3	1.608	VV	0.0773	43.95671	8.10042	3.2647
4	1.748	VV	0.0926	14.72439	2.18640	1.0936
5	1.946	VV	0.1196	29.09728	3.34190	2.1610
6	2.244	VV	0.1337	33.88317	3.35726	2.5165
7	2.496	VV	0.0850	1087.22021	195.11391	80.7476
8	2.687	VB	0.0930	19.19083	2.98476	1.4253
9	3.143	BV	0.2230	35.86568	2.28714	2.6637
10	3.413	VB	0.1252	7.98008	9.57534e-1	0.5927
11	4.060	BB	0.1267	2.79334	3.30273e-1	0.2075
12	4.417	BV	0.1375	6.50855	7.06515e-1	0.4834
13	4.781	VB	0.1438	28.48560	3.02598	2.1156
Totals :				1346.44216	228.55770	

```
    ======================================================================
```

Methyl (2-azido-3,6-di-*O*-benzyl-2-deoxy-4-hydroxy-α-D-glucopyranosyl-(1→4)-(methyl 2-*O*-benzoyl-3-*O*-benzyl-α-L-idopyranosyl) uronate)-(1→4)-2-azido-3,6-di-*O*benzyl-2-deoxy-α-D-glucopyranosyl-(1→4)-(methyl 2-*O*-benzoyl-3-*O*-benzyl-α-L-idopyranosyl)uronate)-(1→4)-6-*O*-acetyl-2-azido-3-*O*-benzyl-2-deoxy-α-D-glucopyranosyl-(1→4)-(methyl 2-*O*-benzoyl-3-*O*-benzyl-α-L-idopyranoside) uronate (O4 deprotection of 8). LCMS 2.

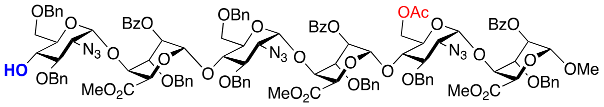

Display Report

Analysis Info

Analysis Name	MB 05-11 GMA6.D	Acquisition Date	16/02/2015 11:38:04
Method	100ACN.M	Operator	mib
Sample Name	MB 05-11/GMA6	Instrument	LC-MSD-Trap-SL
Comment	MB 05-11/GMA6<br>Kinetex XB C18 4.6x100mm 5u<br>100ACN 1ml/min +ve APCI		

Acquisition Parameter

Ion Source Type	APCI	Ion Polarity	Positive	Alternating Ion Polarity	off
Mass Range Mode	Extended	Scan Begin	500 m/z	Scan End	3000 m/z
Capillary Exit	211.0 Volt	Skim 1	40.0 Volt	Trap Drive	170.7
Accumulation Time	200000 µs	Averages	7 Spectra	Auto MS/MS	off

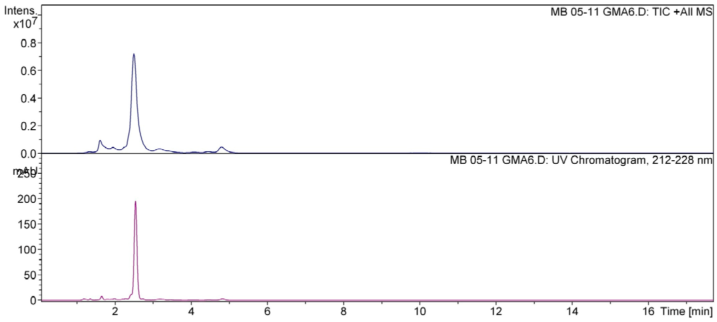

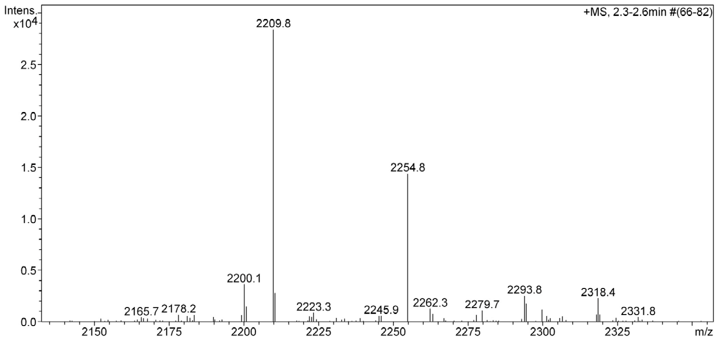

Methyl (6-*O*-acetyl-2-azido-3-*O*-benzyl-2-deoxy-4-*O*-*p*-methoxy-*m*-iodo-benzyl-α-glucopyranosyl-(1→4)-(methyl 2-*O*-benzoyl-3-*O*-benzyl-α-L-idopyranosyl) uronate)-(1→4)-2-azido-3,6-di-*O*-benzyl-2-deoxy-α-D-glucopyranosyl-(1→4)-(methyl 2-*O*-benzoyl-3-*O*-benzyl-α-L-idopyranosyl) uronate)-(1→4)-2-azido-3,6-di-*O*-benzyl-2-deoxy-α-D-glucopyranosyl-(1→4)-(methyl 2-*O*-benzoyl-3-*O*-benzyl-α-L-idopyranosyl)uronate)-(1→4)-6-*O*-acetyl-2-azido-3-*O*-benzyl-2-deoxy-α-D-glucopyranosyl-(1→4)-(methyl 2-*O*-benzoyl-3-*O*-benzyl-α-L-idopyranoside) uronate (9)

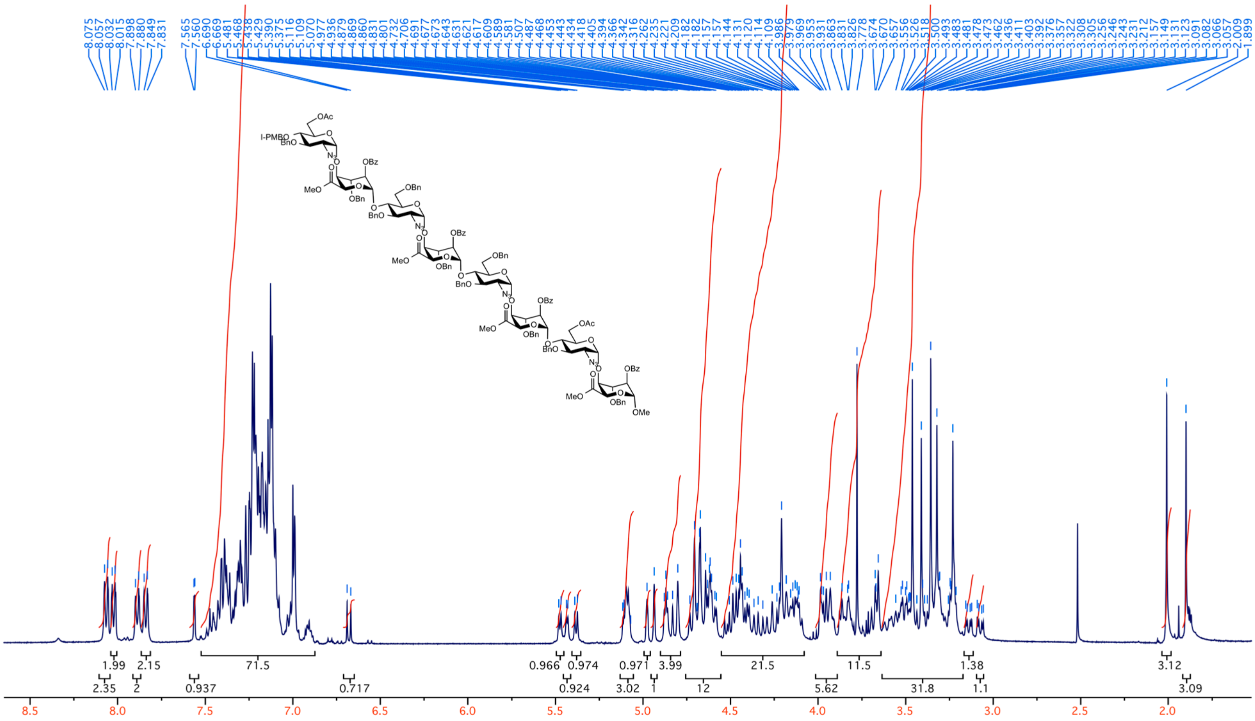

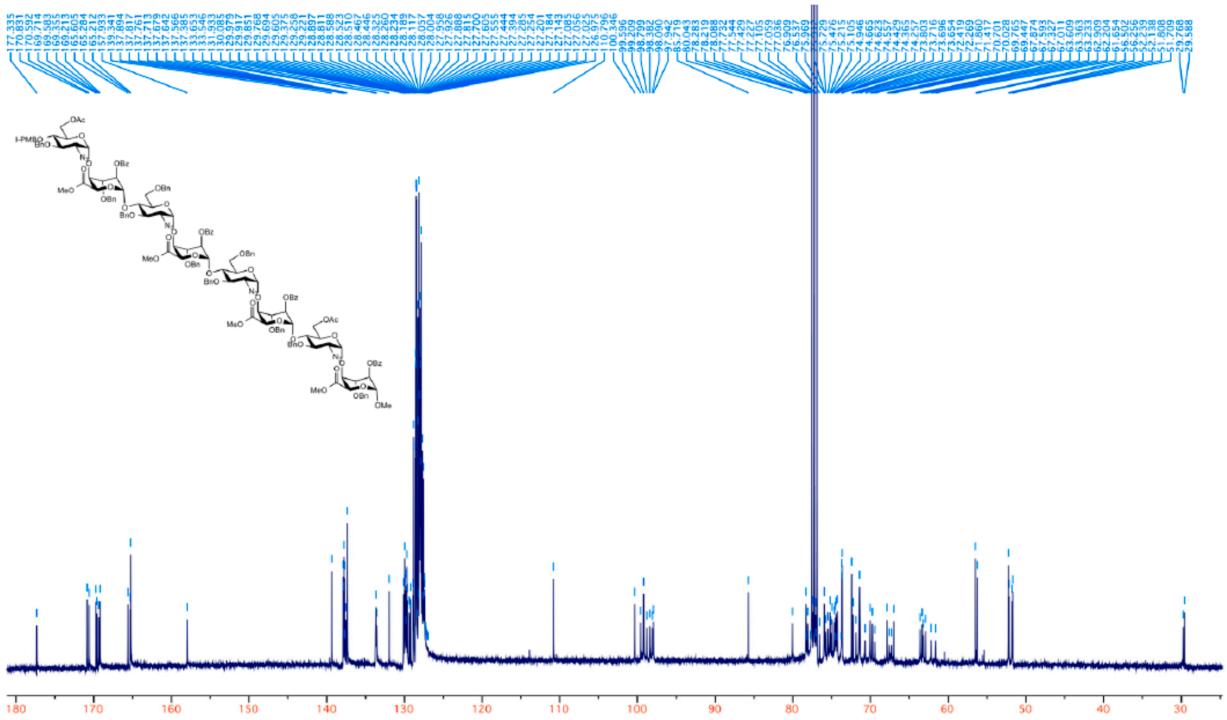

Methyl (6-*O*-acetyl-2-azido-3-*O*-benzyl-2-deoxy-4-*O*-*p*-methoxy-*m*-iodo-benzyl-α-glucopyranosyl-(1→4)-(methyl 2-*O*-benzoyl-3-*O*-benzyl-α-L-idopyranosyl) uronate)-(1→4)-2-azido-3,6-di-*O*-benzyl-2-deoxy-α-D-glucopyranosyl-(1→4)-(methyl 2-*O*-benzoyl-3-*O*-benzyl-α-L-idopyranosyl) uronate)-(1→4)-2-azido-3,6-di-*O*-benzyl-2-deoxy-α-D-glucopyranosyl-(1→4)-(methyl 2-*O*-benzoyl-3-*O*-benzyl-α-L-idopyranosyl)uronate)-(1→4)-6-*O*-acetyl-2-azido-3-*O*-benzyl-2-deoxy-α-D-glucopyranosyl-(1→4)-(methyl 2-*O*-benzoyl-3-*O*-benzyl-α-L-idopyranoside) uronate (9)

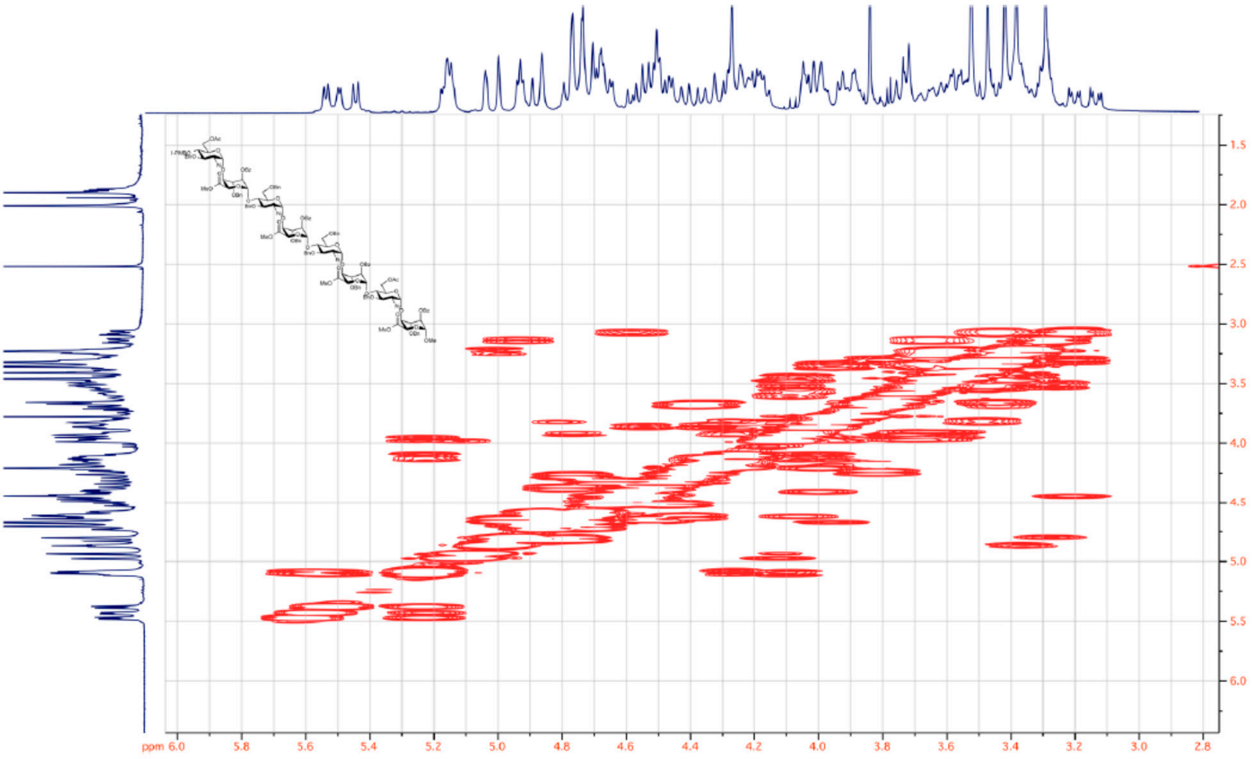

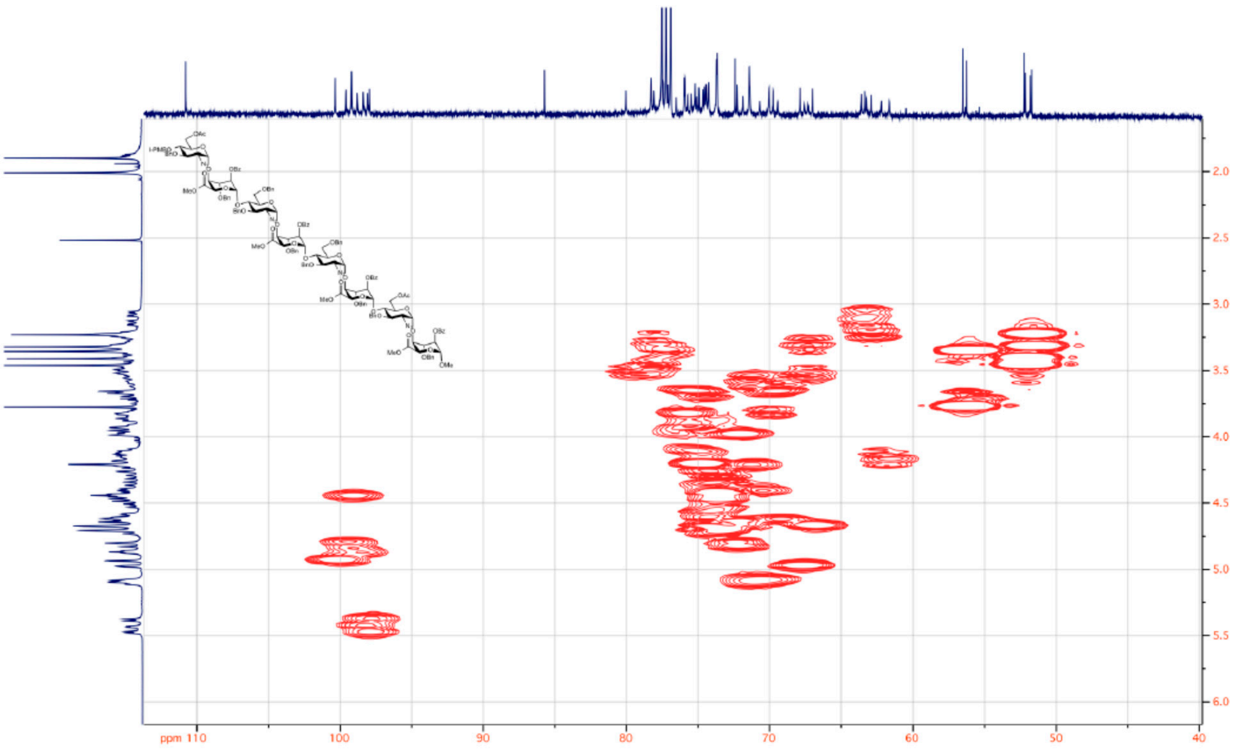

Supplement: Supplementary file 1 [file molecules-20-06167-s001.pdf]
